# The In Situ Electrochemical−Chemical Cascade Strategy for Pyruvic Acid Synthesis

**DOI:** 10.1002/advs.77880

**Published:** 2026-09-27

**Authors:** Xueli Mei, Huawei Zhuo, Yu Liu, Lili Wang, Yudai Huang, Hongtao Xie, Yizhao Li

**Affiliations:** ^1^ State Key Laboratory of Chemistry and Utilization of Carbon Based Energy Resources, College of Chemistry Xinjiang University Urumqi People's Republic of China; ^2^ Huzhou Key Laboratory of Smart and Clean Energy, Yangtze Delta Region Institute (Huzhou) University of Electronic Science and Technology of China Huzhou People's Republic of China

**Keywords:** catalytic conversion of lactic acid, electrosynthesis of pyruvic acid, hydrogen peroxide, two‐electron oxygen reduction reaction

## Abstract

Hydrogen peroxide (H_2_O_2_), as a green oxidizing medium, can be generated and utilized in situ via an electrochemical–chemical cascade, enabling organic transformations while enhancing efficiency and selectivity. However, balancing high H_2_O_2_ production, efficient activation, and selective substrate conversion remains challenging. Herein, a series of transition metal‐doped CeO_2_ catalysts were developed for electrocatalytic H_2_O_2_ generation and its subsequent use in converting α‐hydroxy acids to pyruvate. Among them, Ni−CeO_2_ exhibited the best performance, achieving a pyruvate production rate of 19.9 mmol g^−1^ h^−1^ and a Faradaic efficiency of 55.3%. Mechanistic studies revealed that transition metal doping regulates the local coordination environment and oxygen vacancy concentration, thereby optimizing the adsorption strength of the *OOH intermediate, which governs the two‐electron oxygen reduction reaction pathway and H_2_O_2_ generation and release. Furthermore, in situ generated H_2_O_2_ was selectively activated on the catalyst surface to form a reactive oxygen species network dominated by hydroxyl radicals (·OH) and assisted by singlet oxygen (^1^O_2_). Among these, ·OH showed the lowest energy barrier for α‐C─H bond activation and subsequent electron rearrangement, making it the key species for pyruvate formation. This work provides a new strategy for the preparation of value−added chemicals.

## Graphical abstract

By employing an “unsaturation−oxygen coordination engineering” strategy, the local coordination environment is regulated to achieve efficient electrochemical synthesis of H_2_O_2_. Importantly, an electrochemical−chemical coupled cascade strategy is utilized for the efficient preparation of pyruvic acid via lactic acid oxidation.

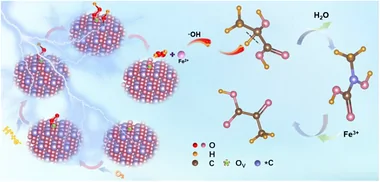

## 1 Introduction

Pyruvic acid is a crucial platform molecule with extensive applications in fields such as food, medicine, agriculture, and bioenergy. It also serves as a key intermediate and metabolic hub in various biochemical reactions. Therefore, the development of efficient and sustainable pyruvate production strategies has long been an important topic in chemistry and chemical engineering [1, 2, 3]. At present, the industrial production of pyruvate mainly relies on biological fermentation and enzymatic catalysis. Although biological fermentation offers advantages such as renewable feedstocks and environmental friendliness, it requires stringent sterile conditions and process control, while also suffering from metabolic inhibition, complex separation and purification procedures, and high overall costs. Enzymatic catalysis, although highly selective and operable under mild conditions, is still limited by catalytic efficiency and long‐term stability [4, 5, 6, 7]. Therefore, the development of green and efficient non−biological conversion pathways is of great significance for overcoming the limitations of existing processes.

In recent years, cascade reactions for the synthesis of value‐added chemicals have attracted increasing attention. For α‐hydroxy acids, selective oxidation can directly produce the corresponding α‐keto acids; thus, the conversion of lactate to pyruvate can serve as a representative model system. Electrocatalytic oxidative upgrading of biomass platform molecules provides a sustainable route for the production of high‐value‐added oxygenated chemicals, in which substrate adsorption, reactive oxygen species generation, and electrode material properties play important roles in determining reaction selectivity [8, 9, 10]. Hydrogen peroxide (H_2_O_2_) [11, 12, 13, 14], as a green oxidant, can be generated in situ through the electrocatalytic two‐electron oxygen reduction reaction (2e‐ORR). Compared with conventional oxidation systems using externally added H_2_O_2_, the in situ generation strategy can avoid the transportation, storage, and purification of H_2_O_2_, while enabling the continuous supply and immediate utilization of the oxidant during the reaction process, thereby providing a new approach for the green and selective oxidation of organic molecules [15, 16]. However, most current studies on H_2_O_2_ electrosynthesis mainly focus on the efficient generation and accumulation of H_2_O_2_ itself, while the correlations among its in situ activation, ROS evolution, and subsequent selective conversion of organic substrates within the same reaction system remain insufficiently understood [17, 18, 19, 20]. The selective generation of H_2_O_2_ via 2e‐ORR and its further efficient conversion into ·OH are two key steps that determine the efficiency of cascade oxidation [21]. Therefore, how to effectively couple electrocatalytic H_2_O_2_ generation, controllable activation, and organic oxidation is a key issue in constructing an efficient electrochemical−chemical cascade system. However, the efficient coupling of H_2_O_2_ generation with selective oxidation remains intrinsically challenging. On the one hand, in situ generated H_2_O_2_ is prone to disproportionation or further reduction, limiting its utilization efficiency. On the other hand, the reactive oxygen species (ROS) generated during H_2_O_2_ activation typically exhibit complex and uncontrolled evolution behavior, and competition among different ROS pathways often leads to nonselective oxidation, thereby limiting the selective formation of target products. Therefore, achieving synergistic regulation among H_2_O_2_ generation, ROS evolution, and organic substrate conversion is the key scientific issue in constructing efficient cascade systems.

Ceria (CeO_2_) is considered an ideal platform for regulating H_2_O_2_ activation and ROS evolution due to its reversible Ce^3+^/Ce^4+^ redox couple, abundant oxygen vacancies (O_V_), and excellent oxygen‐migration capability [22, 23]. In recent years, studies on rare‐earth‐based ORR catalysts have shown that the unique electronic structures, defect‐regulation capability, and interfacial electronic effects of rare‐earth elements can significantly influence oxygen adsorption, electron transfer, and ORR pathway selection [24]. On this basis, introducing transition metal single‐atom sites is expected to further modulate the local coordination environment and electronic structure, thereby enabling control over the adsorption behavior of key intermediates and ROS generation pathways [25, 26, 27, 28, 29, 30]. Previous studies have shown that regulating the local coordination environment of atomically dispersed metal sites can effectively modulate the adsorption behavior of oxygen‐containing intermediates and the ORR pathway selection, providing an important basis for the design of highly selective 2e‐ORR catalysts [31]. However, the intrinsic mechanism by which catalyst local structure influences ROS distribution and subsequently determines organic reaction selectivity remains unclear.

Accordingly, herein we propose an “unsaturated oxygen coordination number” regulation strategy and construct a series of transition metal (Mn, Fe, Co, Ni) single‐atom catalysts on a CeO_2_ support to achieve efficient electrocatalytic H_2_O_2_ generation and its directed utilization in organic transformations. Using lactate as a model substrate, a 2e‐ORR‐organic oxidation cascade system was established to realize the highly selective conversion of lactate to pyruvate. Among these catalysts, Ni−CeO_2_ exhibited the best performance, affording a pyruvate yield of 19.9 mmol g^−1^ h^−1^ with a Faradaic efficiency (FE) of 55.3%. Combined with in situ electron paramagnetic resonance and quenching experiments, hydroxyl radicals (·OH), superoxide radicals (·O_2_^−^), and singlet oxygen (^1^O_2_) were clearly detected in the system. Among them, ·OH played a dominant role in lactate oxidation, whereas ^1^O_2_ exhibited a pronounced synergistic effect. Theoretical calculations further revealed that the decomposition of H_2_O_2_ into ·OH possesses the lowest energy barrier, followed by the ^1^O_2_ pathway. During the subsequent reaction process, the α−C–H activation step involving ·OH exhibited the lowest reaction barrier, thereby dominating the pyruvate formation pathway. These experimental and theoretical results collectively reveal the intrinsic relationship between ROS evolution pathways and reaction selectivity. This work proposes a design principle for directing ROS evolution by regulating the local coordination structure at the mechanistic level, achieving the deep coupling of electrocatalytic in situ H_2_O_2_ generation with the valorization of biomass−derived molecules, and providing important guidance for the integration of electrocatalytic H_2_O_2_ utilization with biomass−derived molecule conversion.

## 2 Results

### 2.1 Characterization of Catalyst Structure

**FIGURE 1:**
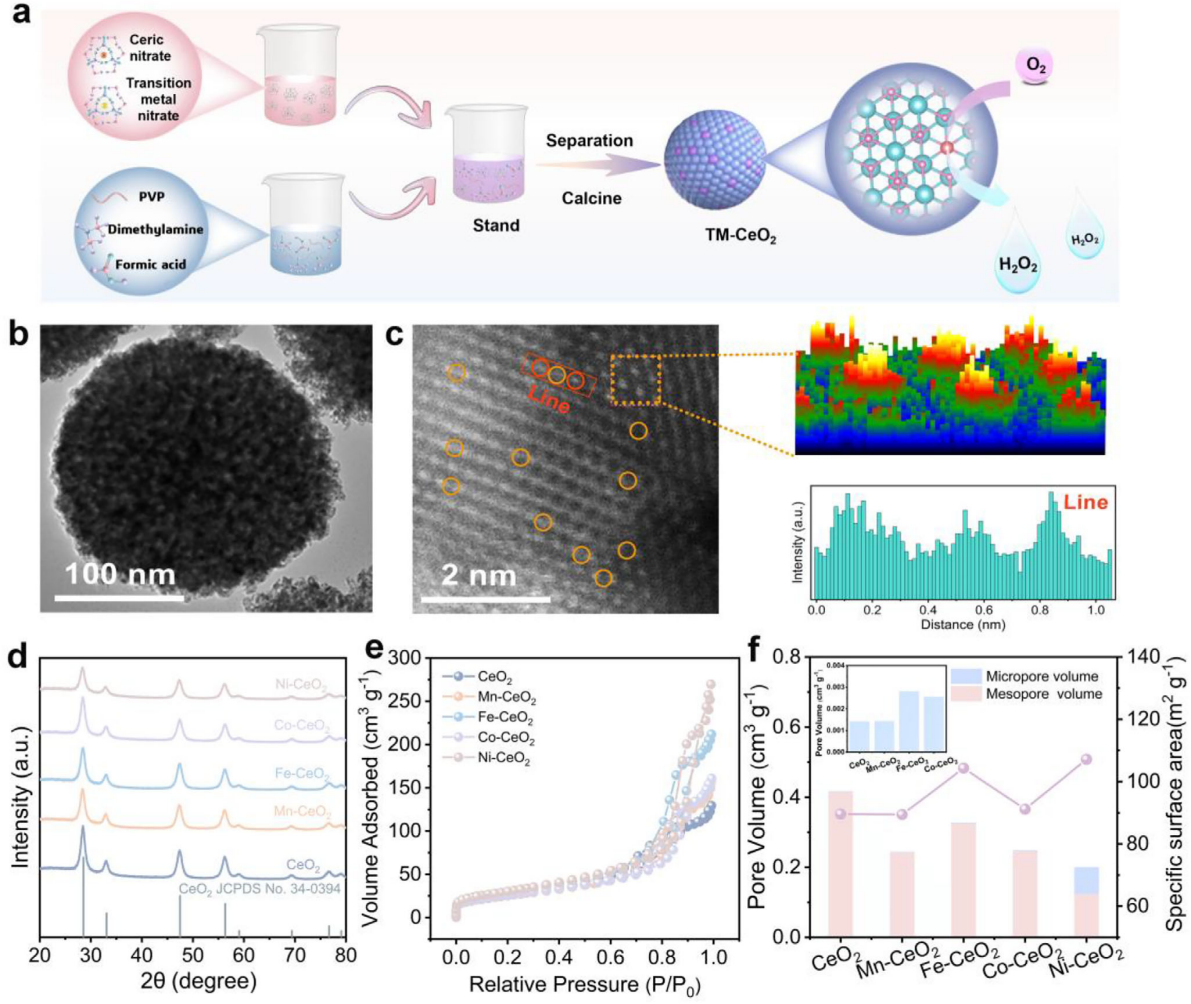
Characterization of catalyst structure. (a) Schematic diagram of the catalyst preparation process; (b) TEM image. (c) AC‐TEM image of Ni−CeO_2_. (d) XRD pattern. (e) N_2_ adsorption−desorption isotherm. (f) Analysis of pore volume and specific surface area of different catalysts.

A series of transition metal‐doped CeO_2_ catalysts (TM−CeO_2_, TM = Mn, Fe, Co, Ni) were successfully synthesized (Figure 1a). All catalysts exhibited uniform spherical nanoparticle morphologies with regular structures and porous surfaces, which are beneficial for mass transfer and the exposure of active sites (Figures 1b and S1,S2). High‐resolution transmission electron microscopy (HRTEM) images clearly revealed the (111) lattice fringes of CeO_2_, and slight changes in the lattice spacing after doping indicated that the introduced transition metals induced subtle lattice distortion (Figure S3). In addition, Ce, O, and the doped metals (Mn, Fe, Co, Ni) were uniformly distributed throughout the support without obvious aggregation (Figure S4). Combined with mass spectrometry (ICP−MS) results, the metal contents in all samples were confirmed to be at relatively low levels (Table S1). To further confirm the dispersion state of the active sites, aberration‐corrected transmission electron microscopy (AC‐TEM) was employed to characterize Ni−CeO_2_. The results showed that Ni [32, 33] was anchored on the CeO_2_ matrix in the form of isolated single atoms (as indicated by the yellow circles in the image), and its three‐dimensional elemental mapping further confirmed the atomic dispersion of Ni (Figure 1c).

All samples displayed characteristic diffraction peaks corresponding to the cubic fluorite structure of CeO_2_ (Figure 1d). The slight shift in diffraction peaks after doping indicates that the transition metals were successfully incorporated into the lattice and altered the lattice parameters. In the Raman spectra (Figure S5), the characteristic F2g mode of CeO_2_ appeared at approximately 460 cm^−1^ [34]. After doping, this peak exhibited a slight red shift and broadening, indicating enhanced lattice distortion and increased O_V_ concentration. In addition, Ni−CeO_2_ possessed the highest specific surface area and exhibited a hierarchical micro‐mesoporous structure, whereas the other samples mainly displayed microporous characteristics (Figure 1e and Table S2). This hierarchical pore structure (Figures 1f and S6) not only facilitates the exposure of more active sites but also promotes the rapid transport of O_2_, H_2_O_2_, and organic substrates at the catalytic interface.

To reveal the effect of doping on the surface electronic structure, X‐ray photoelectron spectroscopy (XPS) analysis was conducted (Figure S7a). The survey spectra confirmed the successful incorporation of all transition metal elements into the CeO_2_ support. Ce 3d spectra (Figure S7b) showed the coexistence of Ce^4+^ and Ce^3+^ species [23, 35]. Compared with pure CeO_2_, the Ce 3d peaks of the doped samples shifted toward lower binding energies, indicating electron transfer from the transition metals to CeO_2_ and an increase in the electron density around Ce sites. Correspondingly, the O 1s peaks (Figure S7c) associated with lattice oxygen (O_L_) and O_V_ shifted toward higher binding energies, further suggesting significant electron redistribution in the system. Quantitative analysis of the Ce^3+^ ratio (Figure S7d) showed that transition metal doping markedly increased the Ce^3+^ content, and the variation trend was highly consistent with the O_V_ concentration obtained from electron paramagnetic resonance (EPR) measurements (Figure S7e). In addition, high‐resolution Ni 2p, Mn 2p, Fe 2p, and Co 2p spectra (Figure S7c−f) demonstrated that all transition metals existed in multiple valence states, indicating strong electronic interactions between the metals and the support. Such strong interactions not only stabilize the single‐atom dispersion state but also regulate the local electronic structure, thereby providing a crucial electronic basis for the subsequent reactions.

**FIGURE 2:**
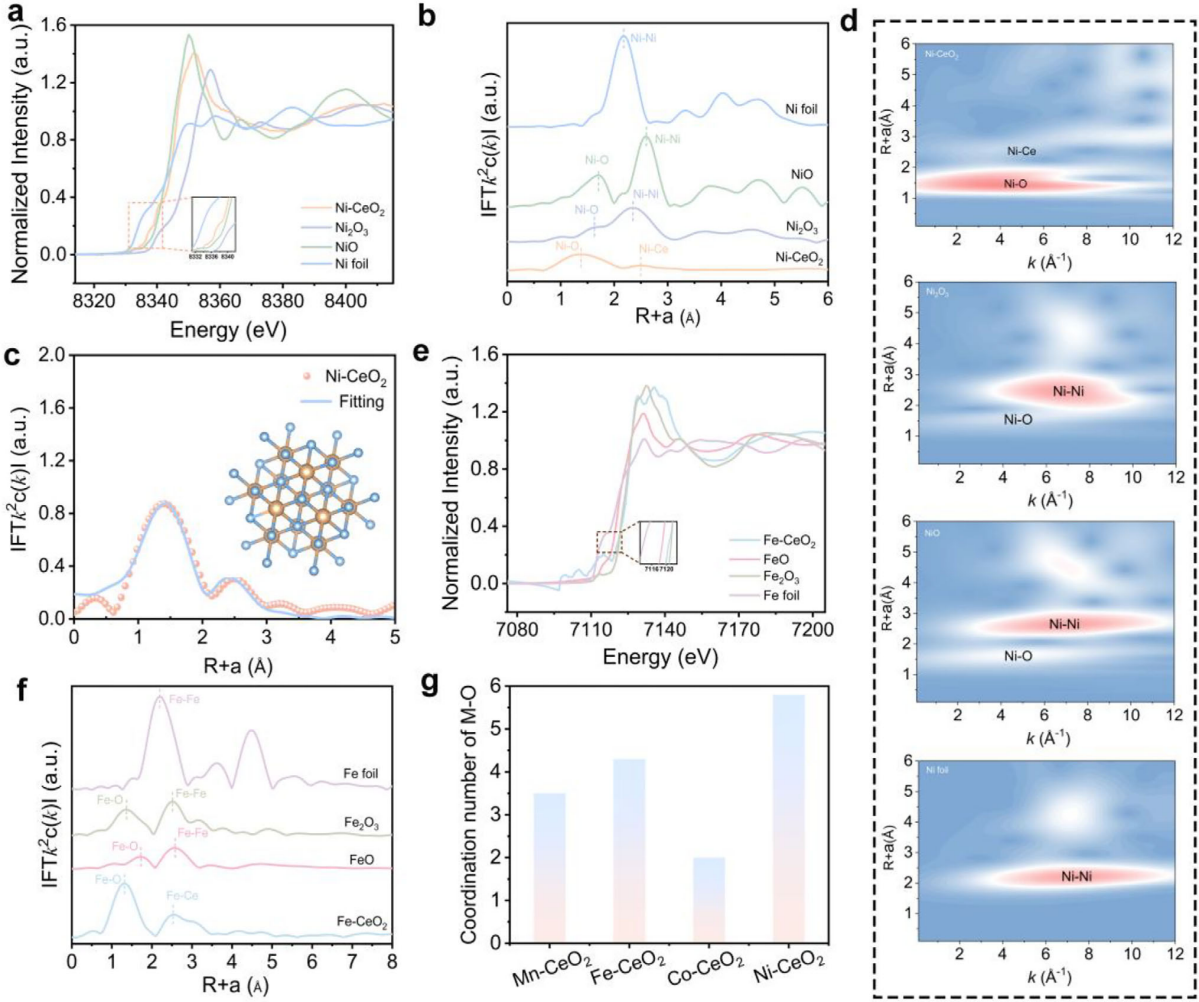
Chemical and electronic structure characterization. (a) Ni K‐edge XANES spectra of Ni−CeO_2_ and reference samples Ni_2_O_3_, NiO, and Ni foil. (b) Corresponding FT‐EXAFS spectra in R space. (c) EXAFS fitting curve of Ni−CeO_2_ in R space. (d) WT−EXAFS of Ni−CeO_2_ and reference samples Ni_2_O_3_, NiO, and Ni foil. (e) Fe K‐edge XANES spectra of Fe−CeO_2_ and reference samples FeO, Fe_2_O_3_, and Fe foil. (f) Corresponding FT−EXAFS spectra in R space. (g) Coordination numbers of Mn−CeO_2_, Fe−CeO_2_, Co−CeO_2_, and Ni−CeO_2_.

X‐ray absorption fine structure (XAFS) analysis was further employed to investigate the atomic‐scale coordination environment. The absorption edge position (Figure 2a) of Ni−CeO_2_ was between those of Ni foil and NiO, closer to NiO, indicating that the average oxidation state of Ni is close to +2. In the Fourier−transformed EXAFS (FT‐EXAFS) spectrum (Figure 2b), only a prominent Ni─O coordination peak was observed at approximately 1.38 Å, while no Ni−Ni scattering signal was detected, confirming the isolated single‐atom dispersion of Ni. Moreover, the Ni─O bond length was determined to be 1.98 Å with a coordination number of approximately 5.8, significantly lower than that of an ideal octahedral coordination environment, indicating a coordinatively unsaturated structure accompanied by local lattice distortion and O_V_ formation [32, 36, 37] (Figures 2c, and S8, and Table S3). Only the Ni−O feature was observed in the wavelet‐transformed EXAFS (WT−EXAFS) spectrum (Figure 2d), further excluding the possibility of metal aggregation. The Fe K‐edge XANES spectrum (Figure 2e) shows that the absorption edge of Fe−CeO_2_ lies between those of FeO and Fe_2_O_3_, approaching Fe_2_O_3_, indicating that Fe predominantly exists in the +3 oxidation state. The FT−EXAFS (Figures 2f, and S9) spectrum reveals an Fe−O bond peak at ∼1.3 Å, with no Fe−Fe bond signal, confirming the atomic dispersion of Fe [37, 38]. K‐edge analyses of Mn and Co (Figures S10, and S11) also confirmed their single‐atom dispersion on the CeO_2_ surface, although their coordination numbers differed significantly (Mn: 3.5, Fe: 4.3, Co: 2.0, Ni: 5.8), reflecting tunable local coordination environments (Figure 2g and Tables S4−S6) [39, 40]. Among them, Co−CeO_2_ exhibited the lowest coordination number and the highest O_V_ concentration, which is consistent with the EPR results. Notably, the degree of local coordinative unsaturation and O_V_ concentration jointly determine the adsorption and activation mode of O_2_. Excessive O_V_ concentrations tend to promote the dissociative adsorption of O_2_, whereas moderately coordinatively unsaturated structures are more favorable for stabilizing molecularly adsorbed intermediates, thereby promoting the 2e‐ORR pathway. These results establish the intrinsic relationship among coordination structure, O_V_, and O_2_ activation mode, providing a critical structural basis for subsequent H_2_O_2_‐selective generation and ROS evolution.

### 2.2 Electrocatalytic 2e‐ORR Performance

To ensure a fair comparison of the catalytic behavior among different transition metal centers, the dopant content of all TM−CeO_2_ catalysts was uniformly fixed at 3%. This value was determined based on a systematic screening of Ni−CeO_2_−X% (X = 1%, 2%, 3%, 5%), in which RRDE results demonstrated that 3% doping delivered the optimal H_2_O_2_ selectivity (Figure S12), thereby excluding the influence of metal loading differences on the reaction pathway. In both 0.01 M and 0.1 M KOH electrolytes, Ni−CeO_2_ exhibited the best activity and H_2_O_2_ selectivity. In 0.1 M KOH, it displayed an onset potential of 0.65 V versus RHE (defined at −0.1 mA cm^−2^) [13], close to the theoretical equilibrium potential (0.7 V vs. RHE), and a Tafel slope of only 51.7 mV dec^−1^, indicating rapid kinetics with nearly zero overpotential (Figure S13a,b). Ni−CeO_2_ achieved an H_2_O_2_ selectivity of 84%−92% within the potential range of 0−0.5 V versus RHE, with an electron transfer number close to 2, clearly following a 2e^−^ reaction pathway (Figure S13c,d). Further Koutecky−Levich (K−L) analysis also showed that the electron transfer number of Ni−CeO_2_ was close to 2, further confirming from a kinetic perspective that it preferentially generates H_2_O_2_ through the 2e‐ORR pathway (Figure S14). Notably, H_2_O_2_ selectivity does not show a simple linear relationship with the oxygen‐vacancy‐related defect concentration, but instead exhibits a distinct defect−window effect (Figure S15 and Table S7). An appropriate amount of oxygen vacancies is beneficial for O_2_ activation and *OOH intermediate formation, whereas excessive oxygen vacancies lead to the over‐adsorption of oxygen‐containing intermediates and promote the competing 4e−ORR pathway. In this TM−CeO_2_ catalyst series, the suitable oxygen‐vacancy‐related defect level is approximately 3.5 × 10^15^ spins g^−1^. Although Mn−CeO_2_ and Ni−CeO_2_ possessed similar electrochemically active surface areas (ECSA) (Figure S16), the latter exhibited significantly higher H_2_O_2_ selectivity, indicating that the reaction pathway is primarily governed by *OOH adsorption behavior rather than the number of active sites. At a current density of 40 mA cm^−2^, the H_2_O_2_ yield of Ni−CeO_2_ reached 3.5 mol g^−1^ h^−1^ (FE = 92.6%) and 1.9 mol g^−1^ h^−1^ (FE = 89.1%) in 0.1 M and 0.01 M KOH, respectively, both of which were markedly superior to those of the other TM‐CeO_2_ catalysts (Figures S17−S23).

**FIGURE 3:**
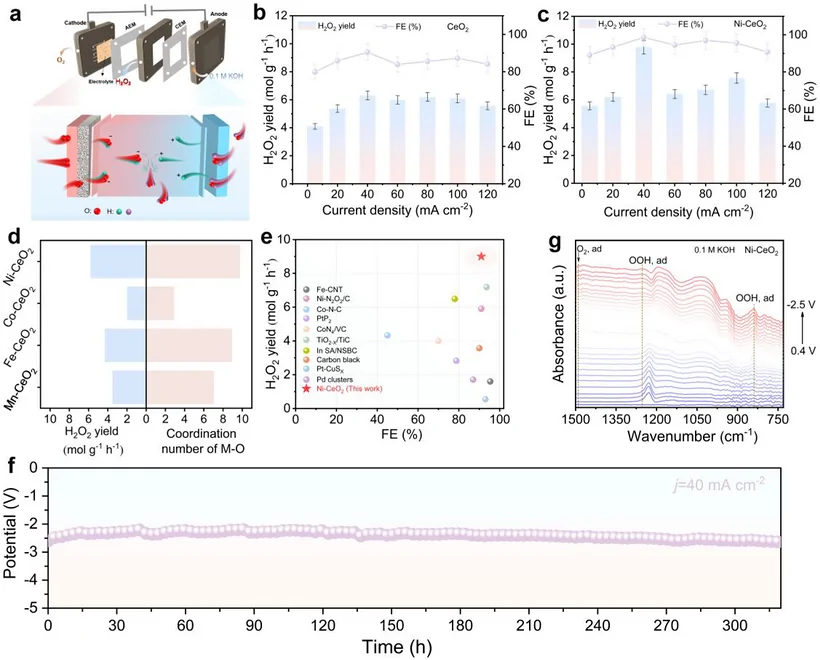
Electrocatalytic 2e‐ORR performance. (a) Schematic diagram of the mechanism for H_2_O_2_ production in a flow cell. In a 0.1 M KOH electrolyte, the H_2_O_2_ yield and FE of (b) CeO_2_ and (c) Ni−CeO_2_ were tested via a flow cell. (d) Correlation between the yield of H_2_O_2_ and the coordination number of M−O. (e) Comparison of the H_2_O_2_ yield and FE of Ni−CeO_2_ with those of other materials (data sources are provided in Table S8). (f) Long‐term cycling stability of Ni−CeO_2_ at 40 mA cm^−2^ current densities. (g) In situ infrared spectra of Ni−CeO_2_.

Considering that O_2_ mass transfer limitations in conventional H‐type cells significantly restrict the H_2_O_2_ yield [41, 42], a flow‐cell reactor was further employed to enhance gas–liquid–solid interfacial mass transfer (Figures 3a and S24). Under conditions of 0.1 M KOH and 40 mA cm^−2^, the H_2_O_2_ yield and FE of Ni−CeO_2_ increased to 9.9 mol g^−1^ h^−1^ and 98.6%, respectively, significantly outperforming the other doped catalysts (Figures 3b,c, S25 and S26). Importantly, the H_2_O_2_ yield showed a positive correlation with the metal–oxygen (M–O) coordination number, indicating that the degree of coordinative unsaturation plays a decisive role in regulating the 2e‐ORR pathway (Figure 3d). The performance of this catalyst surpasses that of most reported systems (Figure 3e), highlighting its remarkable advantage for efficient H_2_O_2_ electrosynthesis. In addition to high activity, catalyst stability is equally important. Under a current density of 40 mA cm^−2^, Ni−CeO_2_ operated continuously for 320 h with almost no voltage decay (Figure 3f), while its morphology and crystal structure remained unchanged (Figures S27 and S28), indicating excellent electrochemical and structural stability as well as good durability over a wide current density range.

In situ attenuated total reflection surface−enhanced infrared absorption spectroscopy (ATR−SEIRAS) was employed to monitor the key intermediates. In both 0.01 M and 0.1 M KOH electrolytes (Figures 3g and S29), the O−O stretching vibration signal of O_2_ was observed at approximately 1490 cm^−1^, indicating that the pre‐adsorption of O_2_ molecules is the initial step of the reaction [43]. In 0.1 M KOH, when the potential decreased below 0.8 V versus RHE, the characteristic peak at approximately 1270 cm^−1^ corresponding to the O−O stretching vibration of the *OOH intermediate and the signal at approximately 834 cm^−1^ assigned to M−O vibrations appeared simultaneously and continuously intensified with further negative potential shift [44, 45]. In contrast, in 0.01 M KOH, these same signals could only be detected at more negative potentials (<1.2 V vs. RHE), indicating that higher alkalinity facilitates the formation of *OOH and accelerates its surface conversion process. In summary, by constructing moderately coordination−unsaturated single−atom sites, Ni−CeO_2_ stabilizes the key *OOH intermediate and efficiently guides the reaction along the 2e‐ORR pathway, achieving highly selective electrochemical H_2_O_2_ synthesis. Additionally, it provides a continuous and controllable source of oxidants for the subsequent selective oxidation of lactic acid to pyruvic acid.

### 2.3 Electrocatalytic Lactate Oxidation and ROS‐Regulated Mechanism

Building upon the efficient in situ H_2_O_2_ electrosynthesis system, the performance of TM‐CeO_2_ catalysts toward the selective oxidation of lactate was further evaluated. Using lactate as the model substrate, an electrochemical–chemical cascade system was constructed in 0.01 M KOH electrolyte to realize the in situ generation and immediate utilization of H_2_O_2_. Considering the decisive role of H_2_O_2_ activation in determining the reaction pathway, different Fe‐based cocatalysts, including ferric citrate, EDTA–Fe, Fe_3_O_4_, and FeCl_2_, were further introduced to promote the conversion of H_2_O_2_ into ROS (Figures S30 and S31). High−performance liquid chromatography (HPLC) analysis showed that FeCl_2_ exhibited the most pronounced promotional effect and significantly enhanced pyruvate production efficiency. This result indicates that Fe^2+^ can efficiently activate the in situ generated H_2_O_2_ and produce ROS through a Fenton‐like process, thereby driving the subsequent oxidation reaction. To exclude the dominant influence of Cl^−^ on the reaction pathway, FeSO_4_ was used as a chloride−free Fe^2+^ source for the control experiment (Figure S32). With the same Fe^2+^ dosage, the FeSO_4_ system could still promote lactic acid conversion and generate pyruvic acid, indicating that pyruvic acid formation mainly originates from the activation of in situ generated H_2_O_2_ by Fe^2+^, rather than depending on the participation of Cl^−^. The performance difference between the FeSO_4_ and FeCl_2_ systems may be related to the influence of different counter anions on the effective state and stability of Fe^2+^. Therefore, FeCl_2_ was employed as the cocatalyst in all subsequent studies.

**FIGURE 4:**
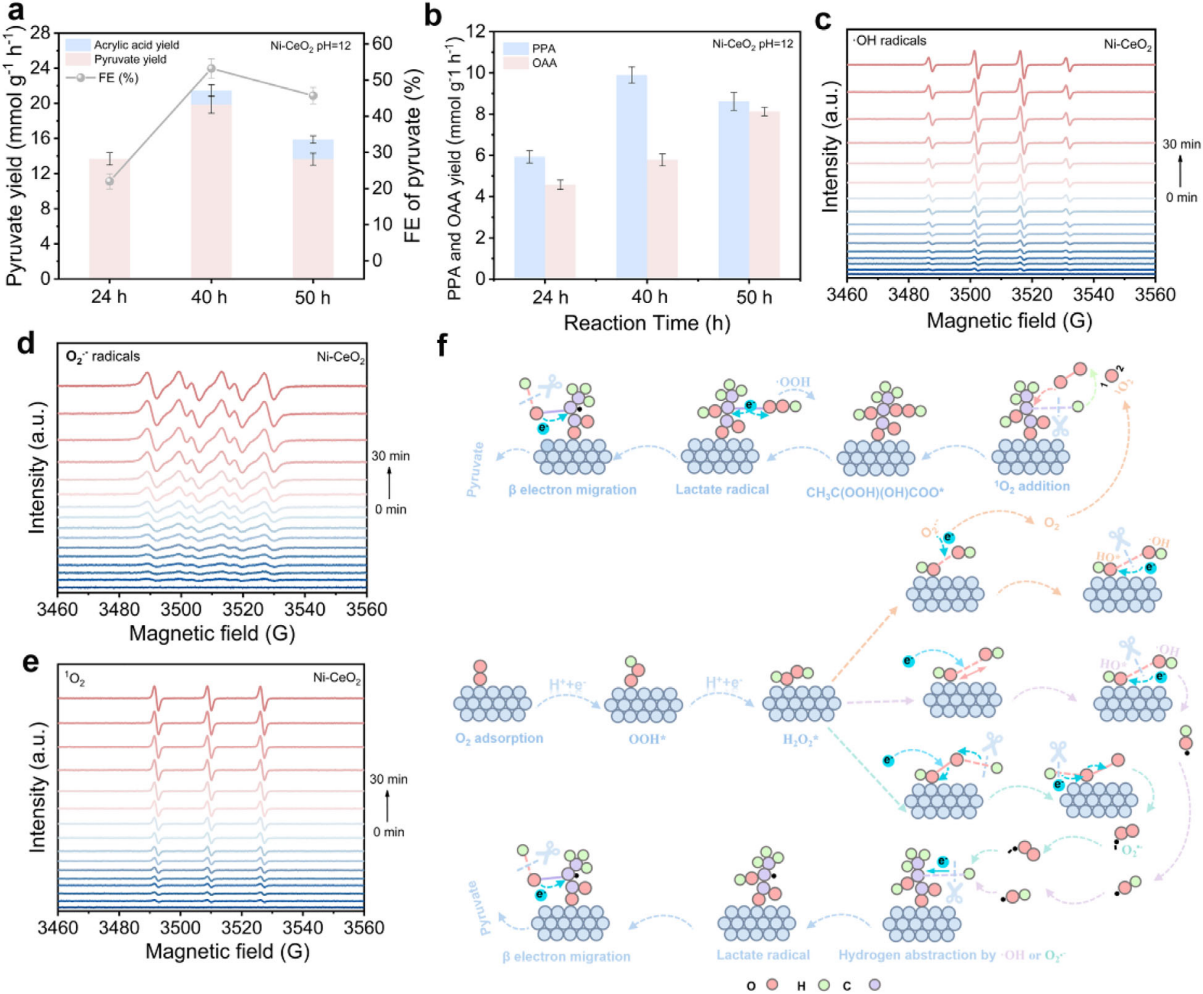
Performance testing of the catalytic conversion of lactic acid to pyruvic acid. (a) The pyruvic acid and acrylic acid yield of Ni−CeO_2_. (b) Yield of PPA and OAA. In situ ESR detection spectra of (c) ·OH, (d) ·O_2_^−^, and (e) ^1^O_2_. (f) Schematic diagram of the mechanism for the conversion of lactic acid to pyruvic acid.

Under optimized conditions, different TM−CeO_2_ catalysts exhibited markedly different catalytic behaviors. Ni−CeO_2_ displayed the best performance, delivering a pyruvate yield of 19.9 mmol g^−1^ h^−1^ with a FE of 55.3%, which was significantly superior to those of CeO_2_ and TM−CeO_2_ (Figures 4a, S33 and S36). This trend is highly consistent with their H_2_O_2_ generation capability in the 2e−ORR process, indicating that the overall efficiency of the cascade system depends not only on the H_2_O_2_ generation rate but also on its subsequent activation behavior. In addition, comparison of the byproduct distribution in the TM‐CeO_2_ catalyst systems showed that, besides the target product pyruvate, a small amount of acrylic acid was also detected, indicating that lactate may undergo competitive dehydration/oxidation side reactions mediated by ROS. Among them, the Fe−CeO_2_ and Ni−CeO_2_ systems produced lower amounts of acrylic acid, with formation rates of 2.4 and 2.2 mmol g^−1^ h^−1^ after 50 h of reaction, respectively, indicating that these two catalysts are more inclined to generate pyruvate through the selective oxidation pathway. In contrast, the acrylic acid formation rates in the Mn−CeO_2_ and Co−CeO_2_ systems reached 3.2 and 3.5 mmol g^−1^ h^−1^, respectively, suggesting that they are more prone to competitive dehydration/oxidation or nonselective side reactions. Due to the low catalytic activity of pure CeO_2_, no obvious byproducts were observed. Further comparison with the acetic acid standard calibration curve showed that no characteristic acetic acid peak was observed in the in situ cascade system, indicating that acetic acid is not a major byproduct in this system (Figure S34). Total organic carbon (TOC) analysis further confirmed that the mineralization rate of the Ni−CeO_2_ system was only 14.7% (Table S9), indicating that it can effectively suppress nonselective oxidation while preferentially maintaining the target product formation pathway.

To further verify the role of the in situ H_2_O_2_ generation strategy, externally added commercial H_2_O_2_ was used as a control instead of in situ electrogenerated H_2_O_2_ (Figure S37). Only a small amount of pyruvic acid was generated in the externally added H_2_O_2_ system, and the yield was significantly lower than that of the Ni−CeO_2_ in situ electrochemical−chemical cascade system. As the concentration of externally added H_2_O_2_ increased, pyruvic acid formation did not increase accordingly; instead, overoxidation‐related byproducts such as acetic acid were more readily detected. This indicates that one‐batch addition of commercial H_2_O_2_ makes it difficult to achieve effective matching among oxidant supply, ROS generation, and selective oxidation of lactic acid, whereas in situ electrogenerated H_2_O_2_ enables continuous supply and immediate utilization, which is more favorable for maintaining the target product formation pathway.

In addition to high activity and selectivity, Ni−CeO_2_ also exhibited excellent stability. After continuous operation for 330 h at 40 mA cm^−2^, almost no decay in operating voltage was observed (Figure S38), and the catalyst morphology and crystal structure remained unchanged before and after the reaction, demonstrating excellent structural stability and resistance to intermediate poisoning (Figures S39 and S40). Further analysis of the Fe and Ni contents in the electrolyte before and after the reaction showed that the Ni content after the reaction was only 0.021 mg L^−1^, indicating extremely low Ni leaching. In contrast, the Fe content decreased from 163.52 to 56.61 mg L^−1^, suggesting that effective Fe species may undergo consumption, deposition, or changes in the Fe^2+^/Fe^3+^ equilibrium during long−term operation (Table S10). Therefore, the slight performance change is more likely related to changes in the effective concentration of Fe species and H_2_O_2_ activation efficiency, rather than damage to the bulk structure of Ni−CeO_2_. Furthermore, to verify the universality of this system, it was extended to other α‐hydroxy acid substrates. When L‐3‐phenyllactic acid and 2‐hydroxybutanedioic acid were used as substrates, phenylpyruvic (PPA) acid and 2‐oxobutanedioic acid (OAA) were generated, respectively, with yields of 9.9 and 8.1 mmol g^−1^ h^−1^ (Figure 4b) and corresponding FE of 27.6% and 22.7% (Figures S41−S45 and Table S11). Compared with lactic acid, L‐3‐phenyllactic acid contains a bulky phenyl substituent, which may weaken its effective adsorption and mass transfer at the catalytic interface and reduce the efficiency of ROS attack on the α‐C−H site; therefore, its product formation rate and Faradaic efficiency decreased. In contrast, 2‐hydroxybutanedioic acid contains two carboxyl groups, which may form relatively strong coordination interactions with Ce sites or oxygen vacancies on the CeO_2_ surface under alkaline conditions, leading to restricted adsorption configurations or slower product desorption, thereby reducing the subsequent oxidation efficiency. These results indicate that this system has preliminary applicability to some representative α‐hydroxy acid substrates, but the molecular size, number of functional groups, adsorption behavior, and difficulty of α‐C−H activation of different substrates significantly affect their reactivity and selectivity.

To reveal the intrinsic mechanism of selective lactate oxidation, the generation behavior and role of ROS during the reaction were systematically investigated. In situ EPR results showed that ·OH, O_2_^−^, and ^1^O_2_ were gradually generated, and their signal intensities continuously increased as the reaction proceeded (Figure 4c−e). Furthermore, radical concentrations in the TM−CeO_2_ systems were measured after 50 h of reaction, and the results indicated that the concentrations of ·OH, ·O_2_^−^, and ^1^O_2_ were all the highest in the Ni−CeO_2_ system (Figure S46 and Table S12). To further distinguish the specific roles of different ROS species, targeted quenching experiments were performed (Figures S47−S50). Upon the addition of isopropanol (IPA) as a ·OH scavenger, the pyruvate yield decreased by 83.5%, indicating that ·OH is the dominant reactive species driving lactate oxidation. When p−benzoquinone (BQ) was added to quench ·O_2_^−^, the yield decreased by only 17.7%, suggesting that its contribution is relatively limited. In contrast, the addition of 1,4−diazabicyclo [2.2.2] octane (DABCO) to suppress ^1^O_2_ led to a 73.5% decrease in pyruvate yield. Although this inhibitory effect was weaker than that of ·OH, it still had a significant influence, indicating that ^1^O_2_ plays an important synergistic role in the reaction.

Based on the above results, the in situ generated H_2_O_2_ was converted into ·OH, ·O_2_^−^, and ^1^O_2_ in the presence of Fe^2+^. These reactive species subsequently interacted with lactate and underwent a series of intermediate evolution and electron rearrangement processes, ultimately leading to pyruvate formation (Figure 4f). Therefore, the Ni−CeO_2_ catalyst successfully established a coupled mechanism spanning in situ H_2_O_2_ generation, ROS evolution, and reaction pathway regulation, thereby constructing a reactive oxygen network dominated by ·OH with the synergistic participation of ^1^O_2_.

### 2.4 Theoretical Insights Into Structure‐ROS‐Selectivity Correlation

**FIGURE 5:**
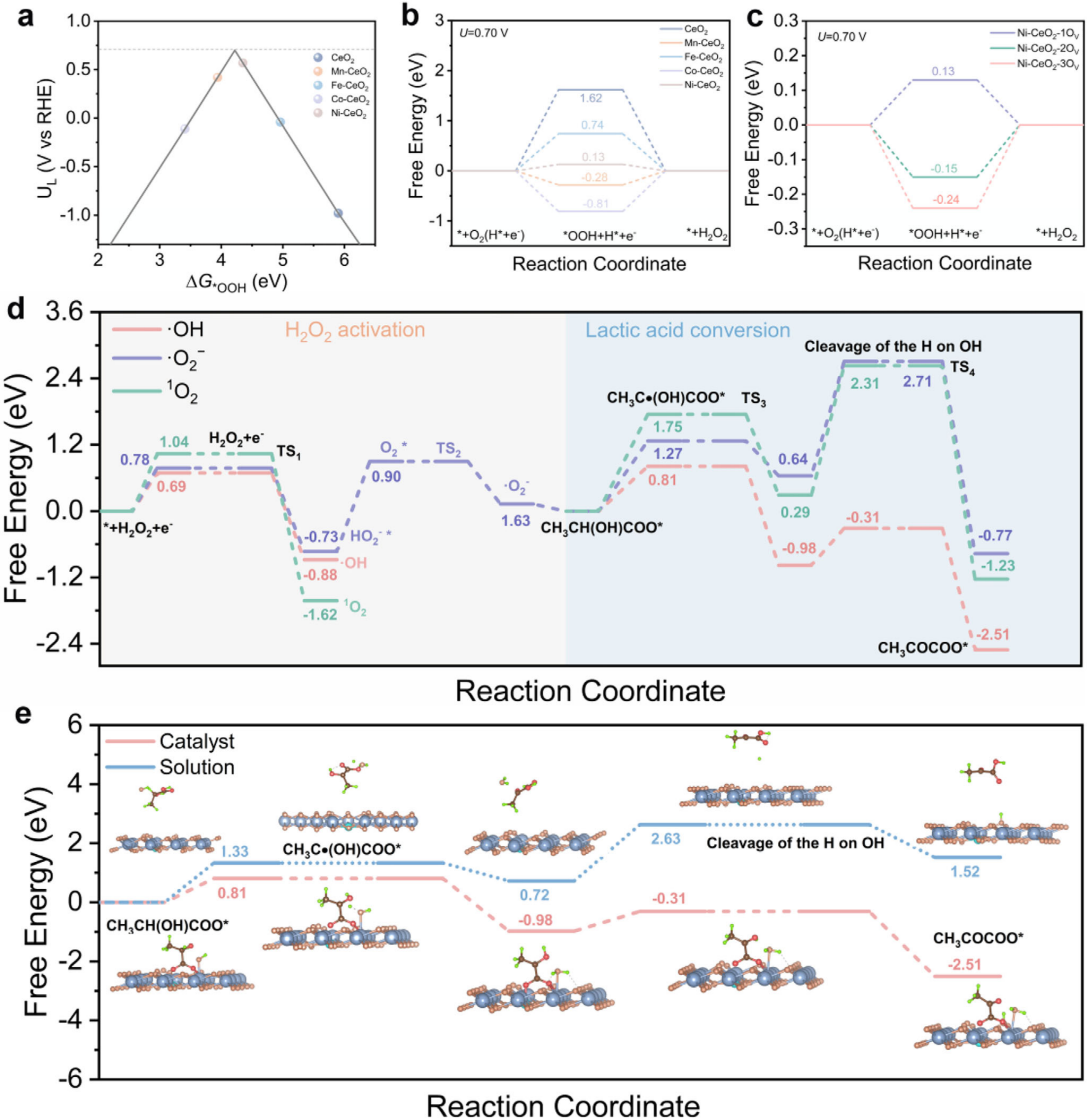
Mechanism analysis. (a) Volcano plot for 2e‐ORR. (b) Free energy diagram for 2e‐ORR at a potential of 0.7 V versus RHE. (c) Free energy diagrams for 2e‐ORR on Ni−CeO_2_ catalysts with different O_V_ concentrations. (d) Free‐energy barrier diagram for the activation of H_2_O_2_ to generate different free radicals and for the catalytic conversion of lactic acid to pyruvic acid. (e) Energy barrier diagram for the adsorption of lactic acid on the catalyst and its subsequent reaction with hydroxyl radicals in solution to form pyruvic acid.

To reveal the regulatory mechanism of catalyst structure on reaction pathways at the atomic scale, density functional theory (DFT) calculations were employed to systematically investigate the 2e‐ORR process and its intrinsic correlation with subsequent selective oxidation of lactic acid (Figures S51−S53). The adsorption free energy of the *OOH intermediate (ΔG_*OOH_) was used as a descriptor to construct a volcano plot for 2e‐ORR activity (Figure 5a). The ΔG_*OOH_ value of Ni−CeO_2_ (4.35 eV) was closest to the theoretical value (∼4.22 eV), achieving an optimal balance between reaction activity and H_2_O_2_ selectivity. Pure CeO_2_, with an excessively high ΔG_*OOH_ (4.57 eV), restricted the formation of O_2_ → *OOH, exhibiting a high reaction energy barrier (1.62 eV) at 0.7 V versus RHE, thereby resulting in low activity. In contrast, Mn−CeO_2_ and Co−CeO_2_, due to overly strong *OOH adsorption, tended to promote O−O bond cleavage, favoring the 4e^−^ pathway to generate H_2_O and significantly reducing H_2_O_2_ selectivity (Figure 5b).

Combining XAFS and EPR results, it was further clarified that the differences in ΔG_*OOH_ originated from changes in the local coordination environment and O_V_ concentration induced by transition metal doping. Although O_V_ could promote O_2_ adsorption and activation, an excessively high O_V_ concentration led to excessive stabilization of *OOH, thereby inhibiting H_2_O_2_ desorption. Therefore, achieving a moderate degree of coordination unsaturation was crucial for maintaining the 2e‐ORR pathway. To further quantify the regulatory role of O_V_, Ni−CeO_2_ models with different O_V_ concentrations, including single, double, and triple O_V_ structures (Ni−CeO_2_, Ni−CeO_2_−2O_V_, Ni−CeO_2_−3O_V_), were constructed (Figures 5c, S54 and S55). The results showed that as the O_V_ concentration increased, ΔG_*__OOH_ decreased to −0.15 and −0.24 eV, respectively, indicating significantly enhanced adsorption compared to the single O_V_ model. This suggested that excessive O_V_ led to excessive stabilization of *OOH, hindering its desorption and weakening 2e‐ORR selectivity, further demonstrating that a moderate O_V_ concentration was key to balancing O_2_ activation and H_2_O_2_ release.

From the perspective of electronic structure, crystal orbital Hamiltonian population (COHP) analysis revealed the decisive influence of metal–oxygen bond strength on *OOH adsorption behavior (Figure S56). The Co/Mn−CeO_2_ systems exhibited high −ICOHP values (−1.25/−1.239 eV), indicating stronger M 3d–O 2p hybridization, which resulted in overly strong *OOH adsorption and inhibited desorption. In contrast, Ni/Fe−CeO_2_ (−0.817/−0.827 eV) demonstrated moderate bond strength, facilitating a dynamic balance between adsorption and desorption. Pure CeO_2_, with weaker bonding (−0.789 eV), struggled to effectively stabilize reaction intermediates, leading to limited activity [45, 46]. Partial density of states (pDOS) analysis further showed that transition metal doping significantly narrowed the bandgap of CeO_2_ and increased the electron density near the Fermi level (Figures S57 and S58) [47, 48], thereby enhancing conductivity and promoting charge transfer processes. Among them, Ni−CeO_2_ exhibited a moderately increased electron density after introducing a single O_V_, which was beneficial for O_2_ adsorption and *OOH formation. However, under high O_V_ concentrations, local electrons significantly accumulated in the Ni–O_V_ region, significantly enhancing *OOH adsorption. Differential charge density analysis visually revealed this electronic regulatory mechanism (Figures S59 and S60). The single O_V_ structure formed a moderate electron‐rich region, facilitating the maintenance of *OOH adsorption–desorption balance. In contrast, under high O_V_ concentrations, excessive electrons localized between Ni and O_V_, leading to overly strong *OOH adsorption and inhibiting the 2e‐ORR pathway.

On this basis, theoretical analysis was further extended to the subsequent lactic acid oxidation process. In this process, the radical generation capacity and its hydrogen abstraction ability from the α‐C−H bond were key steps determining the reaction rate and selectivity. H_2_O_2_ generated on the Ni−CeO_2_ surface preferentially decomposed to form ·OH, which had the lowest reaction energy barrier (Figures 5d, S61 and S62). In comparison, the ^1^O_2_ pathway required higher energy, while the formation of ·O_2_^−^ involved a stepwise dehydrogenation process (H_2_O_2_ → OOH → O_2_^−^) with multiple transition states, making it kinetically unfavorable. This trend was highly consistent with radical quenching experiment results, further indicating that ·OH was the dominant reactive oxygen species in the system. For the subsequent lactic acid oxidation process, lactic acid preferentially adsorbed on Ce sites near O_V_ and reacted with reactive oxygen species in the adsorbed state (Figures 5d and S63−S66). ·OH had the lowest reaction energy barrier for attacking the α‐C−H bond of lactic acid, making it the most likely to initiate lactic acid activation. In contrast, ·O_2_^−^ and ^1^O_2_ corresponded to higher reaction energy barriers. Although ·O_2_^−^ exhibited superior hydrogen abstraction ability from the α‐C−H bond compared to ^1^O_2_, its low concentration in the system due to its kinetically unfavorable generation process limited its overall contribution to the reaction. During the further electron rearrangement of lactic acid radicals and their conversion to pyruvic acid, the ·OH‐participating pathway still had the lowest energy barrier. Further comparison of the reaction behaviors of adsorbed lactic acid and solution‐state lactic acid shows that lactic acid adsorbed on the catalyst surface has a lower α‐C−H activation energy barrier (Figures 5e and S67), indicating that the oxidation of surface‐adsorbed lactic acid is energetically more favorable and may be one of the important reaction pathways for lactate conversion. Considering that Fe^2+^−mediated H_2_O_2_ activation can also generate ROS in the solution phase, the current results more reasonably suggest that both the surface adsorption pathway and the solution‐phase ROS oxidation pathway may jointly participate in this cascade reaction. In summary, a unified structure‐reaction correlation mechanism can be established. Transition metal doping regulates the adsorption behavior of the *OOH intermediate by modulating the local coordination environment and O_V_ concentration, thereby determining H_2_O_2_ generation and release. Furthermore, the in situ generated H_2_O_2_ decomposes on the catalyst surface to form a ROS network dominated by ·OH and synergized by ^1^O_2_, ultimately regulating the reaction pathway and pyruvic acid selectivity of lactic acid.

## 3 Conclusion

In summary, this work has constructed an electrochemical–chemical cascade system based on single‐atom TM−CeO_2_ catalysts, achieving the conversion of O_2_ to H_2_O_2_ and subsequently to the selective oxidation of lactic acid into pyruvic acid. Systematic studies demonstrate that transition metal doping can modify the adsorption behavior of the *OOH intermediate by regulating the local coordination environment and O_V_ concentration, thereby determining the 2e‐ORR pathway and H_2_O_2_ production efficiency. Among them, Ni−CeO_2_ achieves an optimal balance between O_2_ activation and H_2_O_2_ desorption due to its moderate coordination unsaturation structure and O_V_ concentration, thus exhibiting the highest H_2_O_2_ selectivity and production rate. Furthermore, the in situ generated H_2_O_2_ is efficiently activated by Fe^2+^ to form a ROS network dominated by ·OH and synergized by ^1^O_2_. Here, ·OH plays a leading role in activating the α‐C−H bond of lactic acid and facilitating subsequent electron rearrangement, serving as the key reactive species driving pyruvic acid production. Experimental results, together with DFT calculations, collectively indicate that Ni−CeO_2_ can effectively couple H_2_O_2_ generation, ROS evolution, and reaction pathway regulation, ultimately achieving efficient and highly selective oxidation of lactic acid. This work provides a novel design strategy for utilizing electrocatalytically in situ generated H_2_O_2_ to drive the high‐value conversion of biomass platform molecules.

## Author Contributions

**Xueli Mei**: methodology, investigation, data curation, formal analysis, Writing – original draft. **Huawei Zhuo**: investigation, formal analysis. **Yu Liu**: investigation, data curation. **Lili Wang**: data curation. **Yudai Huang**: methodology, funding acquisition, writing – review and editing, resources. **Hongtao Xie**: conceptualization, methodology, writing – review and editing. **Yizhao Li**: conceptualization, methodology, funding acquisition, writing – review and editing, supervision.

## Conflicts of Interest

The authors declare no conflicts of interest.

## Supporting information




**Supporting File**: advs77880‐sup‐0001‐SuppMat.docx.

## Data Availability

The data that support the findings of this study are available from the corresponding author upon reasonable request.
