# Supplementary material for: The In Situ Electrochemical−Chemical Cascade Strategy for Pyruvic Acid Synthesis
Source: Adv Sci (Weinh). 2026 Sep 27:e77880. Online ahead of print. doi: 10.1002/advs.77880 (PMC13616350; doi:10.1002/advs.77880)
Supplement: Supplementary file 1 [file ADVS-9999-e77880-s001.docx]

**Supporting Information**

**The in−situ electrochemical−chemical cascade strategy for pyruvic acid synthesis**

*Xueli Mei^1,2^, Huawei Zhuo^1^, Yu Liu^1,2^, Lili Wang^2^, Yudai Huang^1,*^, Hongtao Xie^2,*^, Yizhao Li^1,2,*^*

^1^State Key Laboratory of Chemistry and Utilization of Carbon Based Energy Resources; College of Chemistry, Xinjiang University, Urumqi 830017, P.R. China.

^2^Huzhou Key Laboratory of Smart and Clean Energy, Yangtze Delta Region Institute (Huzhou), University of Electronic Science and Technology of China, Huzhou 313001, P.R. China.

^*^Corresponding authors. E−mails: huangyd@xju.edu.cn (Y. Huang); xieht@csj.uestc.edu.cn (H. Xie); li[yizhao0809@](mailto:liyizhao0809@126.com)126.com (Y. Li)*.*

**Supplementary Information Contents**

**Experimental Section: number of pages: 82**

**Supplementary Figures: number of figures: 67 (Figure S1-S67)**

**Supplementary Tables: number of tables: 12 (Tables S1-12)**

**Supplementary Notes: 8**

**Contents**

**Experimental Section**

**Chemicals**

Cerium nitrate (Ce(NO_3_)_3_·6H_2_O, >99.9%), cerium sulfate (Ce(SO_4_)_2_·4H_2_O, 99.9%), manganese nitrate (Mn(NO_3_)_2_·4H_2_O, >99.9%), iron nitrate (Fe(NO_3_)_3_·9H_2_O, >99.9%), cobalt nitrate (Co(NO_3_)_2_·6H_2_O, >99.7%), nickel nitrate (Ni(NO_3_)_2_·6H_2_O, >99.9%), methanol (CH_3_OH, 99.5%), dimethylamine ((CH_3_)_2_NH, 99.8%), formic acid (HCOOH, >99.5%), and polyvinylpyrrolidone (PVP K−30) were all purchased from Aladdin Industrial Corporation (Shanghai, China), Teflon−treated carbon fiber paper serving as the support for the gas diffusion electrode (GDE) was provided by Beijing Chemical Reagent Company. Nafion solution (5 wt.%) was purchased from DuPont China.

**Preparation of CeO_2_**

Firstly, 0.667 mmol of Ce(NO_3_)_3_·6H_2_O was dissolved in 30 mL of methanol to prepare solution A. Separately, 5 mmol of HCOOH, 5 mmol of (CH_3_)_2_NH, and 0.5 g of the surfactant PVP K−30 were mixed and dissolved in 30 mL of methanol to obtain solution B. Subsequently, Solution A was added to solution B, and the mixture was stirred uniformly and then allowed to stand for 12 h. The resulting precipitate was washed with methanol and centrifuged three times, followed by drying to obtain a white precursor powder. Finally, the precursor powder was placed in a muffle furnace and heated to 500^o^C at a heating rate of 5 5 ^o^C min^−1^ under an air atmosphere, and calcined at this temperature for 3 h to obtain CeO_2_.

**Preparation of TM−CeO_2_**

When preparing Solution A, in addition to adding 0.667 mmol of Ce(NO_3_)_3_·6H_2_O, 0.02 mmol each of manganese nitrate Mn(NO_3_)_2_·4H_2_O, Fe(NO_3_)_3_·9H_2_O, Co(NO_3_)_2_·6H_2_O, and Ni(NO_3_)_2_·6H_2_O were also dissolved in 30 mL of methanol. The remaining steps were exactly the same as those described above.

**Catalyst characterization**

The morphology of the catalyst was observed using a scanning electron microscope (SEM, GeminiSEM 300) and a transmission electron microscope (TEM, FEI−TALOS−F200X). The phase structure of the catalyst was studied using Raman spectroscopy (Renishaw 1000) and X−ray diffraction spectroscopy (XRD, Bruker D8 Advance). The species, contents, and valence states of elements were characterized using X−ray photoelectron spectroscopy (XPS, Thermo Scientific Nexsa). The content of metal elements in the TM−CeO_2_ samples was investigated using inductively coupled plasma mass spectrometry (ICP−MS, Agilent Technologies 5110). The coordination environment and valence states of atoms were analyzed using X−ray absorption fine structure spectroscopy (XAFS).

**Electrochemical measurements**

The ORR performance of the catalysts was tested by the RRDE. The RRDE parameters used in this paper are as follows: The diameter of the disk electrode is 5.0 mm, with an area of 0.196 cm^2^; the gap between the disk and ring is 0.375 mm; the diameter of the ring is 0.75 mm, and the area is 0.153 cm^2^. 10 mg of catalyst powder was dispersed in a mixture of 800 μL deionized water, 200 μL isopropyl alcohol, and 50 μL Nafion solution (5% wt%), and sonicated for about 1 h to obtain the catalyst ink. The dispersed catalyst ink was taken as 10 μL drops on RRDE, which was dried naturally at room temperature and then electrochemically tested. A three−electrode system was constructed using Hg/HgO and Pt electrodes as reference and counter electrodes respectively by combining them with RRDE. All measured potentials obtained were converted to standard reversible hydrogen electrodes by the equation *E* (vs RHE) = *E* (vs Hg/HgO) + 0.059 × pH + 0.198. The selectivity of H_2_O_2_ and the number of electrons transferred (*n*) can be calculated by the following formula:

$$\text{H}_{\text{2}}\text{O}_{\text{2}}\left( \text{\%} \right)\text{=}\frac{\text{200×}{\text{I}_{\text{r}}}/\text{N}}{\text{I}_{\text{d}}\text{+}{\text{I}_{\text{r}}}/\text{N}}\text{×100\% (1)}$$

$$n=\frac{\text{4×|}\text{I}_{\text{d}}\text{|}}{\text{|}\text{I}_{\text{d}}\text{|}+{\text{I}_{\text{r}}}/\text{N}}\text{ }\text{ (2)}$$

where N is the collection coefficient of the Pt ring, N = 0.217, *I_d_* is the disk current density, and *I_r_* is the ring current density.

**Determination of H_2_O_2_ yield**

Under constant current conditions, the yield of H_2_O_2_ was tested using an H−type cell and a flow cell. To prepare the working electrode, 50 µL of the catalyst ink prepared above was dropped on hydrophilic carbon paper to give a catalyst loading of 0.5 mg cm^−2^. For the flow-cell measurements, a catalyst-coated gas diffusion electrode (GDE) was used as the working electrode. The GDE was prepared by loading the catalyst ink onto hydrophobic carbon paper, with an exposed geometric area of 1 × 1 cm^2^ and a catalyst loading of 0.5 mg cm^−2^. A Pt sheet was used as the counter electrode, and Hg/HgO was used as the reference electrode to establish a three−electrode system. A Nafion membrane was used to separate the cathode and anode compartments. Both the catholyte and anolyte were 0.1 M KOH aqueous solutions. During the flow-cell test, the catholyte was circulated through the cathode compartment at a flow rate of 2 mL min^−1^. 0.01 M KOH and 0.1 M KOH were used as electrolytes, respectively, with the constant current supplied by a workstation (Shanghai Chenhua CHI760E). The concentration of H_2_O_2_ was determined by the cerium sulfate titration method. During this process the yellow Ce^4+^ solution was reduced to colorless Ce^3+^ by H_2_O_2_. Based on the change in Ce^4+^ concentration before and after the reaction, the H_2_O_2_ yield could be estimated. The concentration of H_2_O_2_ is calculated by Ce^4+^ conventional titration according to the following formula:

$$\text{2}\text{Ce}^{\text{4+}}\text{+}\text{H}_{\text{2}}\text{O}_{\text{2}}\text{→2}\text{Ce}^{\text{3+}}\text{+2}\text{H}^{\text{+}}\text{+}\text{O}_{\text{2}}\text{ (3)}$$

$$\text{C}_{(H_{\text{2}}O_{\text{2}})}\text{=}\frac{\text{C}_{\text{0}\text{(}\text{Ce}^{\text{4+}}\text{)}}\text{×}\text{V}_{\text{(}\text{Ce}^{\text{4+}}\text{)}}\text{-}\text{C}_{\left( \text{Ce}^{\text{4+}} \right)}\text{×(}\text{V}_{\text{(Ce}^{\text{4+}}\text{)}}\text{+}\text{V}_{\text{(}\text{H}_{\text{2}}\text{O}_{\text{2}}\text{)}}\text{)}}{\text{2×}\text{V}_{\text{(}\text{H}_{\text{2}}\text{O}_{\text{2}}\text{)}}}\text{ (4)}$$

where *C_0_* (Ce^4+^) and *C* (Ce^4+^) are the concentrations of Ce^4+^ before and after the reaction, *V* (Ce^4+^) and *V* (H_2_O_2_) are the volumes of Ce^4+^ and H_2_O_2_.

To quantify the generated H_2_O_2_ after electrolysis for 1 h, 10 μL of solution in a cathode was added to 4 mL of 0.5 mmol L^−1^ standard Ce(SO_4_)_2_ solution. The mixture solution was measured by UV−Vis after standing for 2 h, and the yield of H_2_O_2_ was finally determined based on the reduced Ce^4+^ concentration. The Faraday efficiency (FE) for H_2_O_2_ generation in H−cells was calculated as follows:

$$\text{FE}\left( \text{\%} \right)\text{=}\frac{\text{Mole of generation}\text{ H}_{\text{2}}\text{O}_{\text{2}}\text{×2×96485}}{\text{Total consumed charge (C)}}\times100\text{ (5)}$$

**Electrocatalytic synthesis of pyruvic acid**

Electrocatalytic synthesis of pyruvic acid was conducted in an H-type electrolysis cell. Typically, 30 mL of 0.01 M KOH containing 0.05 M lactic acid was used as the electrolyte. A Hg/HgO electrode, Pt sheet, and the catalyst-loaded electrode prepared above were used as the reference electrode, counter electrode, and working electrode, respectively. Before electrolysis, 0.09 mmol of FeCl_2_·4H_2_O was added to the electrolyte as an H_2_O_2_ activation cocatalyst. During the reaction, H_2_O_2_ generated in situ through the cathodic two-electron ORR pathway was further activated by Fe^2+^ via a Fenton-like process, producing hydroxyl radicals (·OH) and other reactive oxygen species (ROS) that promoted lactate oxidation to pyruvate. It should be noted that FeCl_2_ was introduced during electrolysis for H_2_O_2_ activation and ROS generation, rather than for post-reaction product separation or purification. At different time points during the reaction, 200 μL of the reaction solution is taken, diluted, and the amount of pyruvate produced is measured using HPLC. Finally, the yield of pyruvate is calculated based on its standard curve. Meanwhile, the mineralization rate of the entire reaction was calculated according to the following formula:

Mineralization rate = $\frac{\text{TOC}_{\text{initial}}\text{-}\text{TOC}_{\text{final}}}{\text{TOC}_{\text{initial}}}$	(6)

**In−situ ATR−SEIRAS**

In−situ attenuated total reflection surface−enhanced infrared absorption spectroscopy (ATR−SEIRAS) measurements were carried out using a Bruker Vertex80V infrared spectrometer fitted with a mercury cadmium telluride (MCT) detector. The procedure for preparing the working electrode is outlined below: First, a gold layer was electrodeposited onto a silicon wafer surface. Subsequently, the catalyst was evenly applied onto the gold−coated silicon wafer. The specific formulation process for the gold plating solution is as follows: (1) Dissolve 0.105 g of NaOH in 67 mL of deionized water, followed by the addition of 3 mL of a 0.1 g mL^−1^ HAuCl_4_ solution. (2) Sequentially dissolve 0.1337 g of NH_4_Cl, 0.6205 g of Na_2_S_2_O_3_·5H_2_O, and 0.9653 g of Na_2_SO_3_ in 30 mL of deionized water. (3) Combine 7 mL of the solution from step (1) with 3 mL of the solution from step (2), and stir the mixture at room temperature until it turns clear and colorless. Before gold plating, the silicon wafer must undergo pretreatment by soaking it in a 40% NH_4_F solution for 90 s, followed by rinsing with water. After pretreatment, blend 2 mL of the gold plating solution with 34 μL of HF, and carefully add this mixture dropwise onto the silicon wafer surface at 55^o^C. Next, rinse the wafer with water and ethanol. Finally, deposit 50 μL of the prepared catalyst ink onto the gold−plated silicon wafer and allow it to air−dry naturally before testing. In−situ ATR−SEIRAS measurements were conducted in a 0.1 M KOH solution, utilizing an Hg/HgO reference electrode and a Pt foil counter electrode.

**Computational details**

All the calculations are performed in the framework of the DFT with the projector augmented plane−wave method, as implemented in the Quantum ESPRESSO [1]. The generalized gradient approximation proposed by Perdew, Burke, and Ernzerhof is selected for the exchange−correlation potential [2]. The long−range van der Waals interaction is described by the DFT−D3 approach [3]. The cut−off energy for the plane wave is set to 450 eV. The energy criterion is set to 10^−5^ eV in the iterative solution of the Kohn−Sham equation. A vacuum layer of 20 Å is added perpendicular to the sheet to avoid artificial interaction between periodic images. The Brillouin zone integration is performed using a 2x2x1 k−mesh. All the structures are relaxed until the residual forces on the atoms have declined to less than 0.02 eV Å^−1^. The computational hydrogen electrode model has been adopted for the calculation of the Gibbs free−energy change for the relevant elemental steps. The free energy was calculated from:

Δ*G* = Δ*E*_DFT_ ＋ Δ*E*_ZPE_ － TΔ*S*	(7)

where Δ*E*_DFT_ is the calculated DFT energy, Δ*E*_ZPE_ and TΔ*S* are obtained from the DFT vibration frequency calculations.

In this work, idealized periodic surface models were used, and the computational hydrogen electrode model was employed to describe the influence of electrode potential on the free energies of the 2e−ORR elementary steps. Explicit water molecules, solvation effects, K^+^ ions, the interfacial electric double layer, and external electric fields were not explicitly included in the current models. In real alkaline electrochemical interfaces, solvation, hydrogen-bonding interactions, surface charge, and the protonation states of reactive species may alter the adsorption free energies and reaction barriers of intermediates. Therefore, the calculated results are mainly used to compare the relative energy trends among different catalyst structures and reaction pathways, and should not be regarded as quantitative predictions of absolute energies or reaction rates under practical electrochemical conditions.

**Supplementary figures**

**Figure S1.** SEM images of (a) CeO_2_, (b) Mn−CeO_2_, (c) Fe−CeO_2_, (d) Co−CeO_2_, and (e) Ni−CeO_2_.

**Figure S2.** TEM images of (a) CeO_2_, (b) Mn−CeO_2_, (c) Fe−CeO_2_, and (d) Co−CeO_2_.

**Figure S3.** HRTEM images of (a) CeO_2_, (b) Mn−CeO_2_, (c) Fe−CeO_2_, (d) Co−CeO_2_, and (e) Ni−CeO_2_.

**Figure S4.** Elemental mapping images of (a) CeO_2_, (b) Mn−CeO_2_, (c) Fe−CeO_2_, (d) Co−CeO_2_, (e) Ni−CeO_2_.

**Supplementary Note 1:** Scanning electron microscopy (SEM) characterization (Figure S1) indicates that all transition metal−doped cerium dioxide (TM−CeO_2_, TM = Mn, Fe, Co, Ni) samples exhibit a highly uniform spherical nanoparticle structure with a concentrated particle size distribution, free from significant aggregation or extraneous phase impurities. This demonstrates that doping with different transition metals does not significantly alter the morphological characteristics of CeO_2_, providing an ideal carrier for the stable dispersion of single−atom sites. Transmission electron microscopy (TEM) images (Figure S2) show that all samples maintain a regular spherical morphology with a porous surface, which is conducive to reactant mass transfer and the exposure of active sites. The doping of Mn, Fe, Co, and Ni does not significantly alter the main structure of CeO_2_, indicating that the doping process has a limited impact on the carrier morphology. High−resolution transmission electron microscopy (HRTEM) images clearly (Figure S3) display the (111) crystal lattice fringes of CeO_2_, with interplanar spacings measured as follows: CeO_2_ (0.336 nm), Mn−CeO_2_ (0.313 nm), Fe−CeO_2_ (0.288 nm), Co−CeO_2_ (0.321 nm), and Ni−CeO_2_ (0.323 nm). The slight contraction of the lattice spacing after doping (approximately 14.3% for Fe−CeO_2_, 6.8% for Mn−CeO_2_, 4.5% for Co−CeO_2_, and 3.9% for Ni−CeO_2_) reflects the subtle modulation effect of transition metals on the lattice structure of CeO_2_, providing a structural basis for regulating oxygen vacancy concentration and catalytic performance. Elemental mapping (Figure S4) analysis further reveals a uniform distribution of Ce, O, and each transition metal element (Mn, Fe, Co, Ni) within the particles, with no aggregation or enrichment observed, confirming the high dispersion of single−atom metals. This uniform distribution effectively enhances the utilization efficiency of active sites, laying the foundation for excellent electrocatalytic performance.

**Figure S5.** Raman spectrum for CeO_2_, Mn−CeO_2_, Fe−CeO_2_, Co−CeO_2_, and Ni−CeO_2_.

**Figure S6.** Average pore size distribution for CeO_2_, Mn−CeO_2_, Fe−CeO_2_, Co−CeO_2_, and Ni−CeO_2_.

**Figure S7.** XPS spectra of CeO_2_, Mn−CeO_2_, Fe−CeO_2_, Co−CeO_2_, and Ni−CeO_2_. (a) XPS full−survey. (b) Ce 3d spectrum. (c) O 1s spectrum. (d) Relative content of Ce^3+^ in CeO_2_ and TM−CeO_2_ samples determined by quantitative analysis of XPS Ce 3d spectra. (e) EPR of CeO_2_ and TM−CeO_2_. (f) Ni 2p spectrum. (g) Mn 2p spectra. (h) Fe 2p spectra, and (i) Co 2p spectra.

**Supplementary Note 2:** High−resolution X−ray photoelectron spectroscopy (XPS) analysis further elucidates the regulatory effects of transition metal doping on the surface chemical states and electronic structure of CeO_2_ catalysts (Figure S7a). The Mn 2p spectrum displays main peaks for Mn 2p_3/2_ (640.6 eV) and Mn 2p_1/2_ (653.5 eV), which, upon peak fitting, are resolved into signals corresponding to multiple valence states of Mn^2+^, Mn^3+^, and Mn^4+^. This indicates that Mn is incorporated into the CeO_2_ lattice in a mixed valence state, forming Mn^2+^/Mn^3+^/Mn^4+^ redox couples that provide active sites for efficient electron transfer (Figure S7g). In the Fe 2p spectrum, the main peaks for Fe 2p_3/2_ (716.7 eV) and Fe 2p_1/2_ (732.8 eV) predominantly correspond to Fe^3+^, accompanied by Fe^2+^ and trace amounts of Fe^0^, demonstrating that Fe exists in a highly dispersed, multivalent state (Figure S6h). Its tunable d−electron configuration effectively reduces the adsorption energy barrier for the *OOH intermediate. In the Co 2p spectrum, the main peak for Co 2p_3/2_ (780.1 eV) reveals the coexistence of Co^2+^ and Co^3+^ upon fitting, further confirming that transition metals are incorporated into the support in an atomically dispersed form (Figure S7i). No peaks associated with metal clusters or nanoparticles are detected in the XPS spectra of all samples, consistent with the results from aberration−corrected electron microscopy.

**Figure S8.** XAFS characterization analysis of the structural properties of Ni−CeO_2_. (a) K−space standard samples of Ni−CeO_2_, Ni_2_O_3_, NiO, and Ni foil. FT−EXAFS curves in R−space and their fitting curves for Ni K−edge of (b) Ni_2_O_3_, (c) NiO, and (d) Ni foil.

**Figure S9.** characterization analysis of the structural properties of Fe−CeO_2_. (a) FT−EXAFS curves in K−space standard samples of Fe−CeO_2_, FeO, Fe_2_O_3_, and Fe foil. FT−EXAFS curves in R−space and their fitting curves for Fe K−edge of (b) Fe−CeO_2_, (c) FeO, (d) Fe_2_O_3_, and (e) Fe foil. (f-i) WT−EXAFS spectra of Fe−CeO_2_ and different reference samples.

**Figure S10.** Characterization analysis of the structural properties of Mn−CeO_2_. (a) Mn K−edge XANES curves. FT−EXAFS curves in (b) R−space and (c) K−space standard samples of Mn−CeO_2_, Mn_2_O_3_, MnO, MnO_2_, and Mn foil. FT−EXAFS curves in R−space and their fitting curves for Mn K−edge of (d) Mn−CeO_2_, (e) Mn_2_O_3_, (f) MnO, (g) MnO_2,_ and (h) Mn foil. (i) WT−EXAFS spectra of Mn−CeO_2_ and different reference samples.

**Figure S11.** AFS characterization analysis of the structural properties of Co−CeO_2_. (a) Co K−edge XANES curves. FT−EXAFS curves in (b) R−space and (c) K−space standard samples of Co−CeO_2_, CoO, Co_3_O_4_, and Co foil. FT−EXAFS curves in R−space and their fitting curves for Co K−edge of (d) Co−CeO_2_, (e) CoO, (f) Co_3_O_4_, and (g) Co foil. (h) WT−EXAFS spectra of Co−CeO_2_ and different reference samples.

**Supplementary Note 3:** Extended X−ray absorption fine structure (EXAFS) analysis provides in−depth insights into the atomic−level coordination environment disparities of Ni, Fe, Mn, and Co in CeO_2_ and their impacts on the oxygen reduction reaction (ORR) pathways. Ni K−edge data (Figure 2a) reveal that Ni is atomically dispersed within the CeO_2_ lattice, with a Ni−O coordination number (5.8 ± 1.2) significantly lower than the standard coordination number of 8 for Ce^4+^. This confirms the presence of oxygen vacancies (O_V_), which are point defects formed by the absence of oxygen atoms in the lattice. Meanwhile, the Ni−O bond length of 1.98 Å, which is shorter than the 2.09 Å observed in NiO, reflects bond contraction and the formation of strong covalent bonds (Table S3). The Fe K−edge data (Figure 2f) reveal an Fe−O peak at approximately 1.3 Å and the absence of an Fe−Fe peak at around 2.2 Å, confirming that Fe is dispersed in a single−atom form. The fitting results indicate that the Fe−O coordination number is 4.3 ± 0.9, which is lower than those in Fe_2_O_3_ (6.5) and FeO (6.0) (Table S4). The Fe−O bond length is 1.91 Å, shorter than the 1.98 Å in Fe_2_O_3_, suggesting that Fe^3+^ substitutes Ce sites, forming a low−coordination distorted structure accompanied by O_V_. Mn−CeO_2_ XANES reveals (Figure S10 and Table S5) Mn oxidation states between +3 and +4, with an extremely low Mn−O coordination number indicating highly dispersed Mn^3+^ and high O_V_ concentration. The Co K−edge XANES (Figure S11a) absorption edge of Co–CeO_2_ is situated between those of the metallic cobalt foil and CoO, indicating that the oxidation state of Co ranges between 0 and +2. The FT−EXAFS spectrum (Figure S11b) displays a prominent Co−O peak solely at approximately 1.4 Å, with no Co−Co signals detected in the 2.2 Å region, confirming that Co is incorporated into the CeO_2_ lattice as isolated single atoms. Quantitative fitting reveals a Co−O bond length of 1.98 Å, which is close to the Co−O bond length (1.91 Å) in Co_3_O_4_. However, the coordination number (Table S6) is only 2.0 ± 0.4, significantly lower than those in CoO (6) and Co_3_O_4_ (4), thus revealing the highest concentration of O_V_.

Comprehensive analysis shows that O_V_ concentration decreases in the order Co−CeO_2_ (CN = 2.0) > Mn−CeO_2_ (CN = 3.5) > Fe−CeO_2_ (CN = 4.3) > Ni−CeO_2_ (CN = 5.8). High O_V_ concentrations promote O_2_ dissociative adsorption, driving the 4e^−^ pathway to produce H_2_O, while moderate concentrations favor molecular O_2_ adsorption, selectively enabling the 2e^−^ pathway to generate H_2_O_2_. Furthermore, metal−oxygen bond strength (bond lengths: Fe−O 1.91 Å, Mn−O 1.92 Å < Ni−O 1.98 Å) and local distortion (Ni σ^2^ > 0.015 Å^2^; Mn/Co σ^2^ < 0.006 Å^2^) synergistically regulate d−orbital degeneracy and π−backbonding intensity: The significant distortion of Ni suppresses O−O bond cleavage, while the high symmetry of Mn/Co accelerates dissociation. Therefore, the selectivity for H_2_O_2_ increases with the length of the M−O bond and structural distortion, while it decreases with the increase in O_V_ concentration. Ni−CeO_2_, with moderate O_V_, the longest M−O bond, and strong local distortion, is expected to be the optimal 2e−ORR catalyst; the extreme O_V_ in Co−CeO_2_ can lead to excessive adsorption of *OOH intermediate, weakening H_2_O_2_ selectivity, a conclusion that will be further validated in subsequent electrochemical and theoretical calculations.

**Figure S12.** In a 0.1 M KOH electrolyte, (a) LSV curves, (b) H_2_O_2_ selectivity, and (c) electron transfer number of CeO_2_, Ni−CeO_2_−1%, Ni−CeO_2_−2%, Ni−CeO_2_−3%, and Ni−CeO_2_−5%.

**Figure S13.** In a 0.1 M KOH electrolyte, (a) LSV curves, (b) Tafel plots, (c) H_2_O_2_ selectivity, (d) Electron transfer numbers of CeO_2_, Mn−CeO_2_, Fe−CeO_2_, Co−CeO_2_, and Ni−CeO_2_.

**Figure S14.** RRDE measurements and Koutecky-Levich analysis of Ni-CeO_2_ for ORR. (a) LSV curves of Ni-CeO_2_ at different rotation speeds from 400 to 2500 rpm in O_2_-saturated electrolyte. (b) Corresponding Koutecky-Levich plots of Ni-CeO_2_ at different potentials.

**Figure S15.** Correlation between the EPR-derived oxygen-vacancy-related spin concentration and H_2_O_2_ selectivity measured at 0.40 V vs RHE for CeO_2_ and TM–CeO_2_ catalysts.

**Figure S16.** The CV curves of (a) CeO_2_, (b) Mn−CeO_2_, (c) Fe−CeO_2_, (d) Co−CeO_2_, (e) Ni−CeO_2_, and (f) C_dl_ values were measured in a 0.1 M KOH solution.

**Figure S17.** The electrochemical performances of the catalysts were evaluated using an RRDE in a 0.01 M KOH electrolyte solution. (a) LSV curves, (b) H_2_O_2_ selectivity, and (c) electron transfer number.

**Supplementary Note 4:** The performances of TM−CeO_2_ catalysts for the 2e−ORR in a 0.01 M KOH solution were evaluated using a rotating ring−disk electrode (RRDE). The catalysts were precisely loaded onto the surface of the disk electrode to catalyze the reduction of O_2_ to H_2_O_2_, while the ring electrode was employed to detect the oxidation current of the generated H_2_O_2_. Linear sweep voltammetry (LSV) tests (Figure S17a) revealed the current responses of both the disk (ORR) and the ring (H_2_O_2_ oxidation). The results demonstrated that Ni−CeO_2_ exhibited the most positive ORR onset potential (0.53 V vs RHE), outperforming CeO_2_ (0.49 V vs RHE), Mn−CeO_2_ (0.50 V vs RHE), Fe−CeO_2_ (0.51 V vs RHE), and Co−CeO_2_ (0.59 V vs RHE), indicating faster ORR reaction kinetics. Additionally, the LSV spectra showed that Ni−CeO_2_ had the highest ring current, suggesting its strongest preference for the 2e−ORR pathway to generate H_2_O_2_. In stark contrast, Co−CeO_2_ displayed the highest disk current and the lowest ring current, indicating its greater inclination toward the four−electron ORR pathway (4e−ORR) for direct H_2_O production. Furthermore, curves depicting the variation of H_2_O_2_ selectivity and electron transfer number (*n*) with electrode potential were plotted for different catalyst samples (Figure S17b−c). Within the potential range of 0−0.45 V vs RHE, Ni−CeO_2_ achieved an H_2_O_2_ selectivity ranging from 22.4%−74.1%, significantly higher than that of CeO_2_ (11.6%−31.3%), Mn−CeO_2_ (13.5%−26.1%), Fe−CeO_2_ (16.9%−46.2%), and Co−CeO_2_ (only 3.4%). The variation in electron transfer number further confirmed that Ni−CeO_2_ exhibited the optimal selectivity and activity for the 2e−ORR pathway among all samples.

**Figure S18.** UV−vis spectra test. (a) The UV−vis absorption spectra of Ce(SO_4_)_2_ at different concentrations in 0.5 M H_2_SO_4_, and (b) the corresponding fitting curve.

**Supplementary Note 5:** The yellow Ce^4+^ solution can undergo reduction to colorless Ce^3+^ through the action of H_2_O_2_ within the electrolyte. The absorbance of Ce^4+^ both before and after the reaction can be precisely quantified using the UV−visible absorption spectrum (UV−Vis), with the measurement wavelength set at approximately 318 nm. Subsequently, Ce^4+^ solutions with varying concentrations (0.1, 0.2, 0.3, and 0.4 mmol L^−1^) were prepared. By employing UV−Vis to analyze these solutions, we obtained their corresponding absorption spectra and established a standard curve (y = 5.1780x + 0.0164, R^2^ = 0.9956).

**Figure S19.** Photograph of the H−type electrolytic cell used in this work.

**Figure S20.** UV−Vis absorption spectra were measured at different current densities in a 0.1 M KOH electrolyte. (a) CeO_2_, (b) Mn−CeO_2_, (c) Fe−CeO_2_, (d) Co−CeO_2_, and (e) Ni−CeO_2_.

**Figure S21.** Yield and FE of (a) CeO_2_, (b) Mn−CeO_2_, (c) Fe−CeO_2_, (d) Co−CeO_2_, and (e) Ni−CeO_2_. in an H−type cell.

**Figure S22.** UV−Vis absorption spectra were measured at different current densities in a 0.01 M KOH electrolyte. (a) CeO_2_, (b) Mn−CeO_2_, (c) Fe−CeO_2_, (d) Co−CeO_2_, and (e) Ni−CeO_2_.

**Figure S23.** The yield and FE of H_2_O_2_ were tested at different current densities using an H−type cell in a 0.01 M KOH electrolyte. (a) CeO_2_, (b) Mn−CeO_2_, (c) Fe−CeO_2_, (d) Co−CeO_2_, and (e) Ni−CeO_2_.

**Supplementary Note 6:** The performances of CeO_2_ and TM−CeO_2_ catalysts for the 2e−ORR were evaluated using an H−type electrolytic cell in a 0.01 M KOH electrolyte. Quantitative analysis was conducted based on the H_2_O_2_ production rate and FE under different constant current densities. At a current density of 40 mA cm^−2^, the H_2_O_2_ yield for CeO_2_, Mn−CeO_2_, Fe−CeO_2_, Co−CeO_2_, and Ni−CeO_2_ were 1.09, 0.95, 1.57, 0.86, and 1.87 mol g^−1^ h^−1^, respectively, with corresponding Faradaic efficiencies (FE) of 69.2%, 65.5%, 72.1%, 63.1%, and 89.1% (Figure S23). The results demonstrated that Ni−CeO_2_ significantly outperformed the other catalysts in terms of both H_2_O_2_ yield and FE, further confirming its superior catalytic performance for the 2e−ORR.

**Figure S24.** Schematic illustration of the flow cell setup used for electrochemical H_2_O_2_ production.

**Figure S25.** The UV−Vis absorption spectra of H_2_O_2_ were tested using a flow cell in a 0.1 M KOH electrolyte. (a) CeO_2_, (b) Mn−CeO_2_, (c) Fe−CeO_2_, (d) Co−CeO_2_, and (e) Ni−CeO_2_.

**Figure S26.** The yield and FE of H_2_O_2_ for (a) Mn−CeO_2_, (b) Fe−CeO_2_, and (c) Co−CeO_2_ in a flow cell using a 0.1 M KOH electrolyte.

**Figure S27.** SEM images of Ni−CeO_2_ before and after a 320 h stability test at a current density of 40 mA cm^−2^: (a) Initial morphology, (b) morphology after the test.

**Figure S28.** XRD pattern of Ni−CeO_2_ after 320 h of stability testing at a current density of 40 mA cm^−2^.

**Figure S29.** In−situ infrared spectra of Ni−CeO_2_ at different potentials under 0.01 M KOH electrolyte.

**Figure S30.** (a) HPLC profile of pyruvic acid. (b) HPLC standard curve of pyruvic acid. (c) HPLC profile of lactic acid. (d) HPLC standard curve of lactic acid.

**Figure S31.** (a−c) represent the HPLC chromatograms of the reaction products from ferric citrate, disodium EDTA, and Fe_3_O_4_, respectively. (d-f) show the yields of pyruvic acid under the corresponding conditions.

**Figure S32**. (a) Time-dependent HPLC chromatograms of the reaction solution using FeSO_4_ as the Fe-based cocatalyst. (b) Pyruvic acid yield and FE at different reaction times.

**Figure S33.** (a) HPLC profile of acrylic acid. (b) HPLC standard curve of acrylic acid.

**Figure S34.** (a) HPLC chromatograms of acetic acid standard solutions with different concentrations. (b) Calibration curve of acetic acid based on the HPLC peak area.

**Figure S35.** HPLC chromatograms of pyruvic acid, lactic acid and acrylic acid at different reaction times. (a) CeO_2_, (b) Mn−CeO_2_, (c) Fe−CeO_2_, (d) Co−CeO_2_, and (e) Ni−CeO_2_.

**Figure S36.** Yield and FE of lactic acid catalytic conversion to pyruvic acid and acrylic acid at different reaction times. (a) CeO_2_, (b) Mn−CeO_2_, (c) Fe−CeO_2_, and (d) Co−CeO_2_.

**Supplementary Note 7:** The efficient catalytic conversion of lactic acid to high−value−added pyruvic acid using H_2_O_2_ generated in situ via electrochemistry not only circumvents the drawbacks of traditional preparation processes but also eliminates the safety hazards associated with H_2_O_2_ transportation. In this study, the performance of CeO_2_ and TM−CeO_2_ in catalyzing the oxidation of lactic acid to pyruvic acid was evaluated in a 0.01 M KOH/FeCl_2_ co−catalytic system (Figure S36). The results demonstrated that Ni−CeO_2_ exhibited the optimal catalytic activity. This can be attributed to its excellent 2e−ORR performance, which enables the generation of a higher concentration of H_2_O_2_ within the same time, subsequently inducing the production of more reactive oxygen species (ROS). Ultimately, this facilitates the efficient and selective conversion of lactic acid to pyruvic acid. Time−dependent studies revealed that during a 50 h continuous electrolysis process, the pyruvic acid yields of all CeO_2_−based catalysts peaked at 40 h of reaction. However, when the reaction time was further extended to 50 h, the yields declined. This was primarily attributed to the continuous decrease in Fe^2+^ concentration in the system, which significantly slowed down the kinetic conversion of H_2_O_2_ to ROS, thereby reducing the efficiency of lactic acid oxidation to pyruvic acid.

**Figure S37.** HPLC analysis of lactic acid oxidation using externally added commercial H_2_O_2_ instead of in situ electrogenerated H_2_O_2_. Time-dependent HPLC chromatograms obtained with different externally added H_2_O_2_ concentrations: (a) 10 mM, (b) 30 mM, (c) 50 mM, (d) 300 mM, and (e) 500 mM. (f) Comparison of pyruvic acid yield at different reaction times under different externally added H_2_O_2_ concentrations.

**Figure S38.** Stability test of Ni−CeO_2_ catalyst in the electrocatalytic oxidation of lactic acid to produce pyruvic acid. Test conditions: H−type electrolytic cell, constant current density of 40 mA cm^−2^.

**Figure S39.** SEM morphological comparison of the Ni−CeO_2_ catalyst before and after electrocatalytic oxidation of lactic acid: (a) Initial catalyst; (b) Catalyst after continuous operation for 330 h at a current density of 40 mA cm^−2^.

**Figure S40.** XRD patterns of the Ni−CeO_2_ catalyst after electrocatalytic oxidation stability testing of lactic acid. Test conditions: Continuous operation for 330 h at a current density of 40 mA cm^−2^.

**Figure S41.** (a) HPLC spectrum of the reaction product phenylpyruvic acid for the Ni−CeO_2_ catalyst; (b) HPLC standard curve of phenylpyruvic acid.

**Figure S42.** HPLC chromatograms of PPA in the reaction solution at different reaction times.

**Figure S43.** (a) HPLC spectrum of the reaction product 2−oxobutanedioic acid for the Ni−CeO_2_ catalyst; (b) HPLC standard curve of the reaction product 2−oxobutanedioic acid.

**Figure S44.** HPLC chromatograms of the reaction product OAA in the reaction solution at different reaction times.

**Figure S45.** Yield of PPA and OAA.

**Figure S46.** After a 50 h reaction in a 0.01 M electrolyte, the concentrations of **a** ∙OH, **b** ·O_2_^−^, and **c** ^1^O_2_.

**Figure S47.** Study on the effect of ·OH radicals on lactic acid conversion. HPLC chromatograms of pyruvic acid and lactic acid under different isopropyl alcohol (IPA) concentrations: (a) 0 mmol, (b) 40 mmol, and (c) 100 mmol.

**Figure S48.** Effect of ·O_2_^−^ on the catalytic conversion of lactic acid and experiments with a concentration gradient of (p−Benzoquinone) BQ: (a) 0 mmol, (b) 0.6 mmol, and (c) 1.0 mmol.

**Figure S49.** Effect of ^1^O_2_ on the catalytic conversion of lactic acid and 1,4−Diazabicyclo [2.2.2] octane (DABCO) concentration gradient: (a) 0 mmol, (b) 10 mmol, and (c) 20 mmol.

**Figure S50.** (a) Pyruvic acid concentrations at different reaction times with the addition of different molar amounts of IPA. (b) Effects of adding different molar amounts of BQ on pyruvate concentrations at various reaction times. (c) Influence of adding different molar amounts of DABCO on pyruvate concentrations over different reaction times.

**Supplementary Note 8:** Under the catalytic action of Fe^2+^, H_2_O_2_ generates various ROS through a Fenton−like reaction. To systematically investigate the impact of these free radicals on the catalytic conversion performance of lactic acid, electron spin resonance (ESR) technology was employed to analyze the solutions of CeO_2_ and TM−CeO_2_ samples after a 50 h reaction in an H−type reactor (Figure S46). The ESR results confirmed the presence of hydroxyl radicals (·OH), superoxide radicals (·O_2_^−^), and singlet oxygen (^1^O_2_) in the reaction solutions. Among them, the concentrations of ·OH, ·O_2_^−^, and ^1^O_2_ in the Ni−CeO_2_ sample were 2.324 x 10^−5^, 1.049 x 10^−5^, and 1.294 x 10^−5^ mol L^−1^, respectively (Table S12), significantly higher than those in other samples, demonstrating its excellent free radical generation capacity.

To further elucidate the roles of these free radicals in the catalytic conversion of lactic acid to pyruvic acid, selective radical scavenging experiments were conducted using specific scavengers. Isopropanol (at concentrations of 0, 40, 80, and 100 mmol) was used to selectively scavenge ·OH radicals. After a 40 h reaction, the pyruvic acid concentrations were 367.1, 103.5, 82.0, and 60.4 µmol L^−1^, respectively (Figure S47 and Figure S50a). The concentration of pyruvic acid significantly decreased after ·OH quenching, indicating that ·OH play a dominant role in the oxidation of lactic acid to pyruvic acid. In contrast, when p−benzoquinone (at concentrations of 0, 0.6, and 1.0 mmol) was used to scavenge ·O_2_^−^, the pyruvic acid concentrations were 367.1, 319.4, and 302.1 µmol L^−1^, respectively, with minimal changes, indicating that ·O_2_^−^ contributes little to this catalytic process (Figure S48 and Figure S50b). Additionally, when 1,4−diazabicyclo [2.2.2] octane (DABCO, at concentrations of 0, 10, and 20 mM) was used to scavenge ^1^O_2_, the pyruvic acid concentrations were 367.1, 135.9, and 97.1 µmol L^−1^, respectively, showing a significant decline (Figure S49 and Figure S50c). This indicated that ^1^O_2_ also plays an important role in the catalytic conversion of lactic acid to pyruvic acid. In summary, during the catalytic oxidation of lactic acid to pyruvic acid by Ni−CeO_2_, ·OH plays a decisive role, ^1^O_2_ exerts a significant synergistic effect, while the contribution of ·O_2_^−^ to key reaction steps is negligible. These findings provide important theoretical insights for optimizing catalyst design and enhancing lactic acid conversion efficiency.

**Figure S51.** The top view and side view of the optimized atomic model (the green circle represents O_V_).

**Figure S52.** The top view and side view of O_2_ intermediate adsorbed on active sites after DFT optimization.

**Figure S53.** The top view and side view of *OOH intermediate adsorbed on active sites after DFT optimization.

**Figure S54.** Top views and side views models of Ni−CeO_2_ with two and three O_V_ after DFT optimization (the green circle represents O_V_).

**Figure S55.** The top view and side view of *OOH intermediate adsorbed on the Ni−CeO_2_−2O_V_ and Ni−CeO_2_−3O_V_ site after DFT optimization.

**Figure S56.** Projected crystal orbital Hamilton populations (pCOHP) for (a) CeO_2_, (b) Mn−CeO_2_, (c) Fe−CeO_2_, (d) Co−CeO_2_ and (e) Ni−CeO_2_.

**Supplementary Note 13:** Projected crystal orbital Hamilton populations (pCOHP) analysis reveals that doping with Mn, Fe, Co, and Ni significantly modulates the distribution of antibonding and bonding orbitals in CeO_2_. As depicted in Figure S56, in Mn−CeO_2_ and Co−CeO_2_, the antibonding orbitals shift to unoccupied states above the Fermi level, effectively weakening the antibonding interactions between Mn/Co and O while enhancing bond strength. The resulting strong M−O bonds inhibit the desorption of the *OOH intermediate and reduce the energy barrier for O−O bond cleavage, thereby significantly promoting the 4e−ORR pathway. In contrast, although the antibonding orbitals in Fe−CeO_2_ and Ni−CeO_2_ partially fall below the Fermi level, their occupancy is markedly reduced. This "weakly occupied" characteristic optimizes the adsorption−desorption equilibrium of *OOH intermediate, thereby favoring selectivity for the 2e−ORR pathway. These differences primarily stem from variations in the 3d electron configurations of the transition metals and their interaction strengths with CeO_2_: The high−spin states of Mn/Co exhibit stronger hybridization with the Ce 4f orbitals, leading to upward shifts in the antibonding states; conversely, the relatively higher electron filling of Fe/Ni results in weaker interactions with CeO_2_, forming partially occupied antibonding states, which ultimately determine the differences in ORR pathway selectivity.

**Figure S57.** PDOS for Ni−CeO_2_ with different O_V_ concentrations: (a) Ni−CeO_2_, (b) Ni−CeO_2_−2O_V_, (c) Ni−CeO_2_−3O_V_.

**Figure S58.** Projected density of states (PDOS) spectra for (a) CeO_2_, (b) Mn−CeO_2_, (c) Fe−CeO_2_ and (d) Co−CeO_2_.

**Figure S59.** Top and side views of differential charge density for CeO_2_, Mn−CeO_2_, Fe−CeO_2_, Co−CeO_2_ and Ni−CeO_2_.

**Figure S60.** Top and side view models of the differential charge density for Ni−CeO_2_ with two and three O_V_.

**Figure S61.** The process of H_2_O_2_ activation to generate ·OH: (a) H_2_O_2_ is adsorbed onto the surface of the catalyst. (b) H_2_O_2_ decomposes into the ·OH–*OH intermediate state. (c) ·OH are ultimately produced.

**Figure S62.** The process of H_2_O_2_ activation to generate ·O_2_^−^: (a) H_2_O_2_ is adsorbed onto the surface of the catalyst. (b) H_2_O_2_ decomposes into the ·OOH–H intermediate state. (c) ·OOH–H undergoes further adsorption. (d) ·OOH–H decomposes into the ·OO–H–H intermediate state. (e) O_2_^−^: are ultimately generated.

**Figure S63.** The activation of H_2_O_2_ to ^1^O_2_: (a) H_2_O_2_ is adsorbed on the catalyst; (b) H_2_O_2_-O_2_ decomposes into the intermediate state of OH-OH-O_2_; (c) ^1^O_2_ and ·OH are generated.

**Figure S64.** The process of ·OH oxidation of lactic acid to generate pyruvic acid: (a) Co-adsorption of the lactate adsorbed species (CH_3_CH(OH)COO*) and·OH. (b) One H atom in CH_3_CH(OH)COO^*^ combines with ·OH, forming an intermediate state with H_2_O. (c) Co-adsorption of CH_3_C(OH)COO^-^ and H_2_O. (d) Intermediate state with the detachment of a H atom from the hydroxyl group. (e) Co-adsorption of the pyruvate adsorbed species (CH_3_C(O)COO^*^), along with H_2_O and H.

**Figure S65.** The process of ·O_2_^−^ oxidation of lactic acid to generate pyruvic acid: (a) CH₃CH(OH)COO*. (b) One H atom in CH_3_CH(OH)COO* combines with a O_2_^−^, forming a ·OOH intermediate state. (c) Co-adsorption of CH_3_C(OH)COO^-^ and ·OOH. (d) Intermediate state with the detachment of a H atom from the hydroxyl group. (e) Co-adsorption of the CH₃C(O)COO*, along with ·OOH and H.

**Figure S66.** The process of ^1^O_2_ oxidizing lactic acid to generate pyruvic acid: (a) CH_3_CH(OH)COO*. (b) One H atom in CH_3_CH(OH)COO* combines with singlet oxygen, forming an intermediate state with H_2_O_2_. (c) Co-adsorption of CH_3_C(OH)COO^-^ and H_2_O_2_. (d) Intermediate state with the detachment of a H atom from the hydroxyl group. e) CH₃C(O)COO* along with co-adsorption of H_2_O_2_ and H.

**Figure S67.** The process of lactic acid being oxidized to pyruvic acid by ·OH in solution: (a) CH_3_CH(OH)COOH and·OH. (b) One H atom in CH_3_CH(OH)COO^-^ combines with ·OH, forming an intermediate state with H_2_O. (c) CH_3_C(OH)COO^-^ and H_2_O. (d) Intermediate state with the detachment of a H atom from the hydroxyl group. (e) CH_3_C(O)COO^*^ and H.

**Supplementary tables**

**Table S1.** The content of Mn, Fe, Co, and Ni elements in TM−CeO_2_.

| Element | Mn | Fe | Co | Ni |
| --- | --- | --- | --- | --- |
| Content (wt%) | 0.52 | 0.65 | 0.95 | 0.7 |

**Table S2.** Specific surface, pore volume and average pore diameter of the prepared samples.

| Samples | Specific surface area (m^2^ g^−1^) | Pore volume (cm^3^ g^−1^) | Average pore diameter (nm) |
| --- | --- | --- | --- |
| CeO_2_ | 92.28 | 0.198 | 7.54 |
| Mn−CeO_2_ | 91.76 | 0.241 | 9.01 |
| Fe−CeO_2_ | 106.87 | 0.326 | 10.99 |
| Co−CeO_2_ | 93.71 | 0.244 | 9.17 |
| Ni−CeO_2_ | 109.79 | 0.416 | 12.53 |

**Table S3.** EXAFS fitting parameters at the Ni K−edge for various samples.

| Sample | Shell | *CN^a^* | *R*(Å)*^b^* | *σ*^2^(Å^2^)*^c^* | Δ*E*_0_(eV)*^d^* | *R* factor |
| --- | --- | --- | --- | --- | --- | --- |
| Ni foil | Ni−Ni | 12.0* | 2.48±0.01 | 0.0055 | −6.3 | 0.0052 |
| NiO | Ni−O | 6.0* | 2.09±0.01 | 0.0072 | −0.8 | 0.0053 |
|  | Ni−Ni | 12.0* | 2.94±0.01 | 0.0067 | −4.6 |  |
| Ni_2_O_3_ | Ni−O | 2.0±0.4 | 1.95±0.01 | 0.0030 | 7.9 | 0.0137 |
|  | Ni−Ni | 6.3±0.5 | 2.63±0.01 | 0.0060 | −2.4 |  |
| Ni−CeO_2_ | Ni−O | 5.8±1.2 | 1.98±0.01 | 0.0150 | −15.0 | 0.0173 |
|  | Ni−Ce | 0.9±0.2 | 2.82±0.01 | 0.0030 | 1.0 |  |

*^a^CN*, coordination number; *^b^R*, distance between absorber and backscatter atoms; *^c^σ*^2^, Debye−Waller factor to account for both thermal and structural disorders; *^d^ΔE*_0_, inner potential correction; R factor indicates the goodness of the fit. S_0_^2^ was fixed to 0.742, according to the experimental EXAFS fit of Ni foil by fixing CN as the known crystallographic value. A reasonable range of EXAFS fitting parameters: 0.700 < Ѕ_0_^2^< 1.000; CN > 0; σ^2^Å^2^＞0; |ΔE_0_|< 10 eV; R factor < 0.02.

**Table S4.** EXAFS fitting parameters at the Fe K−edge for various samples.

| Sample | Shell | *CN^a^* | *R*(Å)*^b^* | *σ*^2^(Å^2^)*^c^* | Δ*E*_0_(eV)*^d^* | *R* factor |
| --- | --- | --- | --- | --- | --- | --- |
| Fe foil | Fe−Fe | 8.0* | 2.47±0.01 | 0.0046 | 5.9 | 0.0058 |
|  | Fe−Fe | 6.0* | 2.85±0.01 | 0.0057 |  |  |
| FeO | Fe−O | 2.0±0.4 | 2.16±0.01 | 0.0070 | 10.0 | 0.0043 |
|  | Fe−Fe | 5.0±1.0 | 3.01±0.01 | 0.0094 | −4.0 |  |
| Fe_2_O_3_ | Fe−O | 6.5±1.3 | 1.98±0.01 | 0.0116 | −2.9 | 0.0147 |
|  | Fe−Fe | 4.5±0.9 | 2.97±0.01 | 0.0043 |  |  |
| Fe−CeO_2_ | Fe−O | 4.3±0.9 | 1.91±0.01 | 0.0030 | −2.6 | 0.0186 |
|  | Fe−Ce | 0.8±0.2 | 3.18±0.01 | 0.0159 | −15.0 |  |

*^a^CN*, coordination number; *^b^R*, distance between absorber and backscatter atoms; *^c^σ*^2^, Debye−Waller factor to account for both thermal and structural disorders; *^d^ΔE*_0_, inner potential correction; R factor indicates the goodness of the fit. S_0_^2^ was fixed to 0.748, according to the experimental EXAFS fit of Fe foil by fixing CN as the known crystallographic value. A reasonable range of EXAFS fitting parameters: 0.700 < Ѕ_0_^2^< 1.000; CN > 0; σ^2^Å^2^＞0; |ΔE_0_|< 10 eV; R factor < 0.02.

**Table S5.** EXAFS fitting parameters at the Mn K−edge for various samples.

| Sample | Shell | *CN^a^* | *R*(Å)*^b^* | *σ*^2^(Å^2^)*^c^* | Δ*E*_0_(eV)*^d^* | *R* factor |
| --- | --- | --- | --- | --- | --- | --- |
| Mn foil | Mn−Mn | 12.0* | 2.68±0.01 | 0.0060 | 7.0 | 0.0165 |
| MnO | Mn−O | 6.0* | 2.21±0.01 | 0.0107 | −1.4 | 0.0045 |
|  | Mn−Mn | 12.0* | 3.13±0.01 | 0.0103 | −4.5 |  |
| Mn_2_O_3_ | Mn−O | 6.0* | 1.92±0.01 | 0.0200 | −10.0 | 0.0182 |
|  | Mn−Mn | 6.0* | 3.14±0.01 | 0.0065 | −0.7 |  |
| MnO_2_ | Mn−O | 6.0* | 1.89±0.01 | 0.0030 | −2.8 | 0.0139 |
|  | Mn−Mn | 4.0* | 2.92±0.01 | 0.0042 | −5.2 |  |
|  | Mn−Mn | 4.0* | 3.63±0.01 | 0.0030 | 4.2 |  |
| Mn−CeO_2_ | Mn−O | 3.5±0.7 | 1.92±0.01 | 0.0030 | 5.9 | 0.0110 |

*^a^CN*, coordination number; *^b^R*, distance between absorber and backscatter atoms; *^c^σ*^2^, Debye−Waller factor to account for both thermal and structural disorders; *^d^ΔE*_0_, inner potential correction; R factor indicates the goodness of the fit. S_0_^2^ was fixed to 0.73, according to the experimental EXAFS fit of MnO_2_ by fixing CN as the known crystallographic value. A reasonable range of EXAFS fitting parameters: 0.700 < Ѕ_0_^2^< 1.000; CN > 0; σ^2^Å^2^＞0; |ΔE_0_|< 10 eV; R factor < 0.02.

**Table S6.** EXAFS fitting parameters at the Co K−edge for various samples.

| Sample | Shell | *CN^a^* | *R*(Å)*^b^* | *σ*^2^(Å^2^)*^c^* | Δ*E*_0_(eV)*^d^* | *R* factor |
| --- | --- | --- | --- | --- | --- | --- |
| Co foil | Co−Co | 12.0* | 2.49±0.01 | 0.0059 | 5.8 | 0.0023 |
| Co_3_O_4_ | Co−O | 4.0* | 1.91±0.01 | 0.0030 | −7.9 | 0.0120 |
| CoO | Co−O | 6.0* | 2.13±0.01 | 0.0100 | 7.9 | 0.0164 |
|  | Co−Co | 8.0* | 2.98±0.01 | 0.0050 | −2.4 |  |
| Co−CeO_2_ | Co−O | 2.0±0.4 | 1.98±0.01 | 0.0056 | −15.0 | 0.0048 |
|  | Co−Ce | 0.9±0.2 | 2.56±0.01 | 0.0096 | 1.0 |  |

*^a^CN*, coordination number; *^b^R*, distance between absorber and backscatter atoms; *^c^σ*^2^, Debye−Waller factor to account for both thermal and structural disorders; *^d^ΔE*_0_, inner potential correction; R factor indicates the goodness of the fit. S_0_^2^ was fixed to 0.81, according to the experimental EXAFS fit of Co foil by fixing CN as the known crystallographic value. A reasonable range of EXAFS fitting parameters: 0.700 < Ѕ_0_^2^< 1.000; CN > 0; σ^2^Å^2^＞0; |ΔE_0_|< 15 eV; R factor < 0.02.

**Table S7.** Oxygen-vacancy-related spin concentrations of CeO_2_ and TM-CeO_2_ catalysts determined by EPR.

| Samples | Oxygen-vacancy-related spin concentration/spins g^−1^ |
| --- | --- |
| CeO_2_ | 3.24 x 10^15^ |
| Mn−CeO_2_ | 3.91 x 10^15^ |
| Fe−CeO_2_ | 3.53 x 10^15^ |
| Co−CeO_2_ | 3.92 x 10^15^ |
| Ni−CeO_2_ | 3.52 x 10^15^ |

**Table S8.** Comparison of performances for Ni−CeO_2_ with other reported 2e−ORR electrocatalysts in alkaline media.

| Catalyst | Electrolyte | Initial potential | Faradaic efficiency | Selectivity | Productivity | References |
| --- | --- | --- | --- | --- | --- | --- |
| Fe−CNT | 0.1 M KOH | 0.82 V (vs RHE) | 95.4% | 95.4% | 1.60 mol g^−1^ h^−1^ | *Nat. Commun.* **10**, 3997 (2019) |
| Ni−N_2_O_2_/C | 0.1 M KOH | 0.84 V (vs RHE) | 45.0% | 91% | 5.9 mol g^−1^ h^−1^ | *Angew Chem. Int. Ed.* **59**, 13057–13062 (2020) |
| Co−N−C | 1.0 M KOH | 0.84 V (vs RHE) | 78.8% | 45% | 4.33 mol g^−1^ h^−1^ | *J. Am. Chem. Soc.* **141**, 12372–12381 (2019) |
| PtP_2_ | 1.0 M KOH | 0.78 V (vs RHE) | 83.0% | 78.8% | 2.83 mol g^−1^ h^−1^ | *Nat. Commun.* **11**, 3928 (2020) |
| CoN_4_/VG | 0.1 M KOH | 0.76 V (vs RHE) | 93.6% | 70% | 4.00 mol g^−1^ h^−1^ | *Energy Environ. Sci.* **15**, 1172–1182 (2022) |
| TiO_2−x_/TiC | 0.1 M KOH | 0.70 V (vs RHE) | 95.4% | 93.6% | 7.19 mol g^−1^ h^−1^ | *ACS Catal.* **11**, 13797–13808 (2021) |
| In SA/NSBC | 0.1 M KOH | 0.73 V (vs RHE) | 78.0% | 78% | 6.49 mol g^−1^ h^−1^ | *ACS Appl. Mater. Interfaces* **13**, 33182–33187 (2021) |
| Carbon black | 0.1 M KOH | 0.75 V (vs RHE) | 90.0% | 90% | 3.57 mol g^−1^ h^−1^ | *Angew Chem. Int. Ed.* **61**, e202117347 (2022) |
| Pt−CuS_x_ | 0.1 M KOH | 0.71 V (vs RHE) | 93.0% | 93% | 0.55 mol g^−1^ h^−1^ | *Science* **366**, 226–231 (2019) |
| Pd clusters | 0.1 M KOH | 0.72 V (vs RHE) | 87.0% | 87% | 1.71 mol g^−1^ h^−1^ | *Nat. Commun.* **11**, 2178 (2020) |
| Ni−CeO_2_ | 0.1 M KOH | 0.70 V (vs RHE) | 96.1% | 92.0% | 9.90 mol g^−1^ h^−1^ | This work |

**Table S9.** The TOC values before and after the reaction and the mineralization rate for the Ni−CeO_2_ catalyst.

|  | TOC/mg/L | Mineralization rate/% |
| --- | --- | --- |
| Before reaction | 1539.5 | 14.71 |
| After reaction | 1313 |  |

**Table S10.** Fe and Ni contents in the electrolyte before and after the 330 h long-term stability test of Ni-CeO_2_ for lactic acid oxidation.

|  | Fe content (mg L^-1^) | Ni content (mg L^-1^) |
| --- | --- | --- |
| Before reaction | 163.52 | ─ |
| After reaction | 56.61 | 0.021 |

**Table S11.** Summary of the yields and FE of pyruvate, phenylpyruvate, and 2−oxobutanoic acid.

|  | Yield/mmol g^−1^ h^−1^ | FE/% |
| --- | --- | --- |
| pyruvic acid | 19.9 | 53.2 |
| phenylpyruvic acid | 9.9 | 27.6 |
| 2−oxobutanedioic acid | 8.12 | 22.7 |

**Table S12.** The concentrations of ∙OH, ·O_2_^−^, and ^1^O_2_ radicals after 50 h of reaction for CeO_2_, Mn−CeO_2_, Fe−CeO_2_, Co−CeO_2_, and Ni−CeO_2_.

| Sample | CeO_2_ | Mn−CeO_2_ | Fe−CeO_2_ | Co−CeO_2_ | Ni−CeO_2_ |
| --- | --- | --- | --- | --- | --- |
| Concentration of ∙OH radicals/mol L^−1^ | 2.324x10^−5^ | 1.923x10^−5^ | 2.766x10^−5^ | 1.614x10^−5^ | 3.424x10^−5^ |
| Concentration of ·O_2_^−^ radicals/mol L^−1^ | 6.563x10^−6^ | 4.584x10^−6^ | 8.421x10^−6^ | 2.985x10^−6^ | 1.049x10^−5^ |
| Concentration of ^1^O_2_ radicals/mol L^−1^ | 7.065x10^−6^ | 4.951x10^−6^ | 1.012x10^−5^ | 3.215x10^−6^ | 1.294x10^−5^ |

**Supplementary References**

1.	G. Kresse, D. Joubert, “From ultrasoft pseudopotentials to the projector augmented-wave method,” *Physical Review B* 59 (1999): 1758–1775.

2	J. P. Perdew, K. Burke, M. Ernzerhof, “Generalized gradient approximation made simple,” *Physical Review Letters* 77 (1996): 3865–3868.

3	S. Grimme, J. Antony, S. Ehrlich, H. Krieg, “A consistent and accurate ab initio parametrization of density functional dispersion correction (DFT-D) for the 94 elements H-Pu,” *The Journal of Chemical Physics* 132 (2010): 154104.
